# Chalcogen Bond Mediated Enhancement of Cooperative Ion‐Pair Recognition

**DOI:** 10.1002/anie.202001125

**Published:** 2020-05-12

**Authors:** Thanthapatra Bunchuay, Andrew Docker, Utt Eiamprasert, Panida Surawatanawong, Asha Brown, Paul D. Beer

**Affiliations:** ^1^ Department of Chemistry University of Oxford Chemistry Research Laboratory Mansfield Road Oxford OX1 3TA United Kingdom; ^2^ Department of Chemistry and Center of Excellence for Innovation in Chemistry (PERCH-CIC), Faculty of Science Mahidol University 272 Thanon Rama VI, Ratchathewi Bangkok 10400 Thailand; ^3^ Department of Chemistry Faculty of Science and Technology Rajamangala University of Technology Thanyaburi Thanyaburi Pathum Thani 12110 Thailand

**Keywords:** chalcogen bonding, cooperativity, halogen bonding, heteroditopic receptors, ion-pair receptors

## Abstract

A series of heteroditopic receptors containing halogen bond (XB) and unprecedented chalcogen bond (ChB) donors integrated into a 3,5‐bis‐triazole pyridine structure covalently linked to benzo‐15‐crown‐5 ether motifs exhibit remarkable cooperative recognition of halide anions. Multi‐nuclear ^1^H, ^13^C, ^125^Te and ^19^F NMR, ion pair binding investigations reveal sodium cation–benzo‐crown ether binding dramatically enhances the recognition of bromide and iodide halide anions, with the chalcogen bonding heteroditopic receptor notably displaying the largest enhancement of halide binding strength of over two hundred‐fold, in comparison to the halogen bonding and hydrogen bonding heteroditopic receptor analogues. DFT calculations suggest crown ether sodium cation complexation induces a polarisation of the sigma hole of ChB and XB heteroditopic receptor donors as a significant contribution to the origin of the unique cooperativity exhibited by these systems.

## Graphical abstract

**Halogen bond and chalcogen bond ion‐pair heteroditopic receptors** exhibit remarkable cooperative recognition of halide anions via sodium cation–benzo‐crown ether binding. The chalcogen bonding receptor displays the largest enhancement of halide binding strength of over two hundred‐fold. DFT calculations suggest sodium cation crown ether complexation induces a polarisation of the ChB and XB donor atoms as the source of cooperativity.

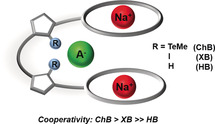

## Introduction

Charged species are ubiquitous, playing crucial roles in biology, medical healthcare and in particular in the environment resulting from polluting anthropogenic chemical industry activities. Cation and anion binding affinities of monotopic receptor systems are critically influenced by the nature of counterions. This has stimulated an ever increasing interest in the construction of heteroditopic receptors for ion‐pair recognition, which are designed to enhance the efficacy of charged guest recognition via favourable intramolecular electrostatic interactions and conformational allosteric cooperativity.^1^, ^2^, ^3^, ^4^ Such systems have been demonstrated to facilitate the solubilisation of inorganic salts in organic media,^5^, ^6^, ^7^, ^8^ to function as efficient extraction and membrane transport reagents,^9^, ^10^ and to be capable of recognising biologically relevant zwitterionic species.^11^, ^12^, ^13^

A heteroditopic receptor typically utilizes Lewis acidic groups and most commonly, various hydrogen bond (HB) donor motifs for recognition of anion guest species.^1^, ^14^ During the last decade or so, the sigma hole bonding interactions halogen bonding (XB) and chalcogen bonding (ChB), which are the attractive non‐covalent interactions between an electrophilic halogen or chalcogen atom covalently linked to an electron‐withdrawing group and a Lewis base, have been exploited in anion supramolecular chemistry. In particular, XB receptors and more recently ChB hosts have been shown to display contrasting and often superior anion binding strength and selectivities in comparison to HB receptor analogues. Taking this into account, it is surprising that the integration of sigma hole donors into heteroditopic structural host framework design is rare. Indeed, to date, there are only three examples of halogen bonding ion‐pair receptors reported^15^, ^16^, ^17^ and to the best of our knowledge, the incorporation of a chalcogen bond donor group into heteroditopic host structures for ion‐pair recognition is unprecedented.

**Figure 1:**
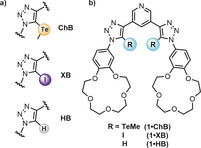
a) The 1,2,3‐triazole donors as the anion binding functionalities. b) Heteroditopic **1⋅XB**, **1⋅ChB** and **1⋅HB** receptor molecules.

Herein, we report a series of heteroditopic receptors containing halogen (**1⋅XB**), chalcogen (**1⋅ChB**), and hydrogen bond (**1⋅HB**) donors integrated into a 3,5‐bis‐triazole pyridine structure for anion recognition which is covalently linked to benzo‐15‐crown‐5 ether motifs for sodium cation binding (Figure 1). Importantly, ion‐pair binding investigations reveal sodium cation–benzocrown ether binding switches on the recognition of bromide and iodide halide anions. Notably, the chalcogen bonding heteroditopic receptor displays a remarkable enhancement of halide binding strength of over two hundred‐fold relative to XB and HB analogues.

## Results and Discussion

### Synthesis of Heteroditopic Receptors

**Scheme 1:**
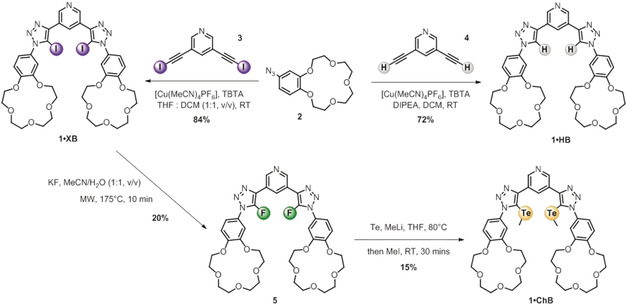
Synthesis of **1⋅XB** and **1⋅HB** via CuAAC reaction and **1⋅ChB** via sequential S_N_Ar reactions.

The target HB and XB heteroditopic receptors were prepared by high yielding copper(I)‐catalysed azide–alkyne cycloaddition (CuAAC) methodology (Scheme 1). The **1⋅XB** receptor was prepared by reaction between two equivalents of 4‐azido‐benzo‐15‐crown‐5 (**2**) and 3,5‐diiodoethynylpyridine (**3**)^18^ in the presence of [Cu(MeCN)_4_]PF_6_ and TBTA in a mixture of 1:1 THF/DCM at room temperature. Recrystallisation from MeOH afforded **1⋅XB** in 84 % yield. The HB analogue was synthesised from 3,5‐diethynylpyridine (**4**) in DCM under the same conditions and purification method to afford **1⋅HB** in 72 % yield (Scheme 1) The preparation of bis‐methyltelluro‐triazole containing ChB receptor (**1⋅ChB**) proved to be much more challenging and was initially attempted by a nucleophilic substitution reaction between in situ generated lithium methyl telluride anion and **1⋅XB**.^19^ Under these conditions, the bis‐iodotriazole (**1⋅XB**) starting material was dehalogenated to the bis‐prototriazole analogue **1⋅HB** with no evidence of formation of the desired product. An alternative synthetic approach was undertaken via the preparation of a bis‐fluorotriazole intermediate (**5**) which was more reactive towards nucleophilic aromatic substitution.^20^ The XB receptor (**1⋅XB**) was treated with KF in acetonitrile/water mixtures under microwave irradiation, and the desired product (**5**) isolated as an orange solid in 20 % yield after chromatographic purification. An S_N_Ar reaction of in situ generated lithium methyl telluride from methyl lithium and tellurium(0) powder with **5** afforded, as evidenced from ESI‐MS analysis, a mixture of products including **1⋅ChB**, **1⋅HB** and the mixed ChB/HB compound analogue. The target chalcogen bonding heteroditopic receptor **1⋅ChB** was successfully isolated in 15 % yield after preparative thin layer chromatographic purification. All three heteroditopic receptors were characterised by ^1^H, ^13^C, ^125^Te NMR (where relevant) and high‐resolution ESI‐MS spectrometry.

### Solution Binding Studies

The ion‐pair binding properties of **1⋅XB**, **1⋅ChB** and **1⋅HB** ditopic receptors were studied by ^1^H NMR spectroscopic titration experiments in 10 % [D_6_]DMSO–CDCl_3_ solvent media. To evaluate quantitative analysis of ion‐pair recognition, comparative titrations were carried out in the presence and absence of sodium cations, the complementary sized alkali metal cation for the benzo‐15‐crown‐5 binding motif. In addition to association constants for anion and ion‐pair binding, cooperativity factors (*α*=*K* ${}_{\mathrm{Na}{}^{+}}$ /*K*_none_) were determined for comparison of XB, ChB and HB enhancement of anion binding that results from cooperative binding of cation and anion in the host. The ^1^H NMR titration experiments of **1⋅XB** and **1⋅HB** with TBA halide salts typically caused a downfield shift of the internal pyridine and phenyl proton resonances in the ditopic receptors. Additionally, the shift of the triazole proton in the HB receptor also indicated its participation in the anion binding event. Perturbation in the chemical shift of the internal pyridine proton incumbent upon the anion binding cavity was observed and monitored as a function of anion concentration. Very small perturbations were observed in analogous halide titrations with **1⋅ChB**. WinEQNMR2 analysis^21^ of the titration data determined 1:1 stoichiometric association constants shown in Table 1. Although the association constant values are modest, notably the **1⋅XB** receptor displays stronger halide binding than the **1⋅HB** and **1⋅ChB** receptors, with no selectivity preference. Interestingly, the **1⋅ChB** host binds all the halides very weakly (*K*_a_<5 m ^−1^) compared to the **1⋅XB** and **1⋅HB** analogues.

**Table 1:** Anion association constants *K*_a_ [m ^−1^] for **1⋅XB**, **1⋅ChB** and **1⋅HB** in the presence and absence of two equivalents of sodium cation in 10 % [D_6_]DMSO–CDCl_3_.

	Anion association constants *K*_a_ [m ^−1^] for heteroditopic acyclic receptors^[a]^								
Anions^[b]^	**1⋅XB**			**1⋅ChB**			**1⋅HB**		
	Na^+[c]^	none	α^[d]^	Na^+[c]^	none^[b]^	α^[d]^	Na^+[c]^	none^[b]^	α^[d]^
Cl^−^	^[e]^	35	^[e]^	^[e]^	<5	–	^[e]^	10	–
Br^−^	5060	40	**127**	1400	<5	**280**	379	18	**21**
I^−^	5660	35	**162**	1080	<5	**216**	364	16	**23**

[a] Association constants calculated using WinEQNMR2 software, errors (±) less than 10 %. [b] Anions added as their TBA salts. [c] Titration conducted in the presence of two equivalents of NaPF_6_. [d] The cooperativity factor (α) is given by *α*={*K*_a_(Na^+^)/*K*_a_(none)}. [e] It was not possible to determine an association constant from the titration data suggesting complex equilibria and/or possible competing precipitation of sodium chloride.

The addition of two equivalents of NaPF_6_ to ^1^H NMR solutions (10 % [D_6_]DMSO–CDCl_3_) of each heteroditopic host did not cause any perturbations of the respective pyridyl host protons which provided evidence for hexafluorophosphate anion not participating in anion complexation. In all cases significant downfield perturbations of the phenyl protons as well as the crown ether methylene protons in the crown ether region were observed indicative of sodium cation binding in each crown ether binding site (Figure S1–S3).

**Figure 2:**
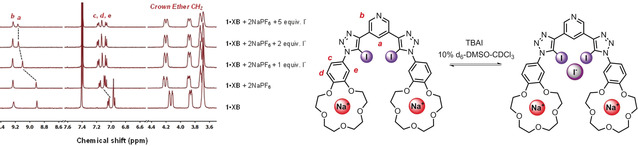
Truncated ^1^H NMR spectra of XB receptor, **1⋅XB** in the presence of 2 equivalents of NaPF_6_ with increasing equivalents of TBAI, and binding equilibrium in 10 % [D_6_]DMSO in CDCl_3_ (500 MHz, *T*=298 K).

The ^1^H NMR halide titrations were then repeated in the presence of two equivalents of NaPF_6._ With all three heteroditopic receptors, a significant downfield shift of the respective internal pyridine (a) and phenyl proton (c,d,e) resonances was observed, whilst the signals associated with the crown ether protons remained unchanged (representative example for **1⋅XB** shown in Figure 2). This implies that halide anion binding occurs within the respective bis‐triazole cleft of the heteroditopic receptor and not simply via direct electrostatic association with the benzo crown ether bound sodium cations. Monitoring the internal pyridine proton (a) of each receptor, association constants were determined from the titration data using WinEQNMR2 (Table 1). In the case of bromide and iodide, the sigma hole type receptors (**1⋅XB** and **1⋅ChB**) display much stronger halide binding compared to the **1⋅HB** analogue; impressively over an order of magnitude greater with **1⋅XB**.^22^

Importantly, Table 1 demonstrates that for each heteroditopic receptor the association constant values for bromide and iodide anions are increased dramatically in the presence of co‐bound sodium cations. The combination of favourable electrostatics and the crown ether bound sodium cation inductively withdrawing electron density from the respective XB, ChB, and HB anion recognition site via a conjugated through‐bond mechanism may be responsible for the observed cooperativity of ion‐pair binding.^23^ It is noteworthy that the calculated cooperativity factors (*α*=*K* ${}_{\mathrm{Na}{}^{+}}$ /*K*_none_) are the greatest for **1⋅ChB**, being at least over a two hundred fold enhancement of halide binding strength, followed by **1⋅XB** over a hundred fold halide binding enhancement, which is an order of magnitude larger than **1⋅HB** (Table 1). In effect, with the chalcogen bonding heteroditopic receptor **1⋅ChB**, the presence of crown ether co‐bound sodium cations switches on the recognition of bromide and iodide anions. The greatly enhanced cooperativity of ion‐pair recognition by the XB and ChB receptors relative to the HB analogue may be rationalised by increased polarisation of C−X (X=I and TeMe) via sodium complexation in the conjugated heteroditopic receptors.

Further evidence for the participation of halogen bonding in **1⋅XB** ion‐pair recognition was also observed by a comparison of ^13^C NMR spectra of **1⋅XB** in the presence of two equivalents of NaPF_6_, and two equivalents of NaI (Figure S4). The chemical shift of the quaternary carbon of the bis‐iodotriazole motif was significantly perturbed upfield with NaI (Δ*δ*=−2.22 ppm). Iodide anion complexation was also confirmed by the upfield shift of internal pyridine carbon (Δ*δ*=−1.45 ppm), which indicates the halide anion being located in the bis‐iodotriazole XB binding cleft of the receptor. Analogous experiments with **1⋅ChB** were carried out to provide evidence for chalcogen bond formation, probed by ^125^Te NMR spectroscopy, between the bis‐methyltelluro triazole motif and iodide. The ^125^Te nucleus, despite its low natural abundance, is highly sensitive to changes in the chemical environment as well as non‐covalent interaction formation in solution.^24^, ^25^ A complexation experiment with two equivalents of NaPF_6_ revealed an upfield perturbation due to crown ether sodium cation complexation. By contrast, addition of two equivalents of NaI resulted in a downfield shift indicative of iodide ChB anion recognition (Figure S5).

The genuine participation of halogen bonding or chalcogen bonding, as opposed to electrostatic contribution was confirmed by a series of control experiments using the pyridine‐bis‐fluorotriazole crown ether host **5** as a model in this study. The fluorotriazole derivative serves as a non‐halogen bond donor group,^26^ where anion coordination is only viably governed by electrostatic interactions. From qualitative ^1^H NMR spectroscopic experiments, the sodium complexed fluorotriazole receptor **5** did not display any proton resonance perturbations upon addition of 5 equivalents of iodide anion, indicating no anion binding to this receptor (Figure S6b).^27^ Moreover, the ^19^F NMR spectrum also indicates no perturbation of the fluorine atom in the fluorotriazole group by iodide anion (Figure S6c). From this experiment, this indicates that the sodium‐complexed bis‐triazole receptors do not assist cooperative ion‐pair binding via simple direct electrostatic interaction between the cations and anion.

### Computational DFT studies

**Figure 3:**
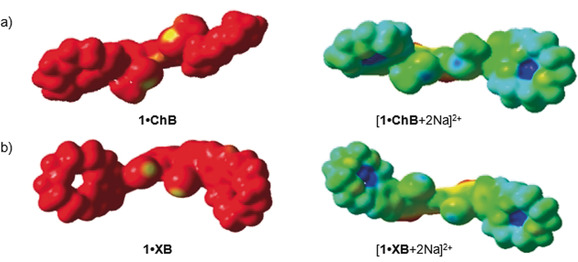
Electrostatic potential plots (ESPs) were mapped over electron density surfaces with an isodensity of 0.004 au with the color scale from 0.05 to 0.26 au for **1⋅ChB** and **1⋅XB** and their sodium complexes.

Having demonstrated the unique ion‐pair recognition properties of **1⋅ChB** and **1⋅XB**, DFT calculations were performed to gain insight into the electronic structures of the sigma hole mediated heteroditopic receptors and importantly the perturbation of that structure upon Na^+^ complexation.^28^ A survey of the calculated binding energies of the free and sodium‐complexed receptors, summarised in Table 2, show good agreement to the experimentally observed trend to the association constants obtained from ^1^H NMR titration experiments. As anticipated from increased electrostatic interactions towards the anionic iodide guest, the dicationic complexes [**1⋅ChB**+2 Na]^2+^ and [**1⋅XB**+2 Na]^2+^ show dramatically enhanced I^−^ association energies relative to the **1⋅ChB** and **1⋅XB**. Indeed, further evidence for the strengthening of the sigma hole–iodide interaction was suggested by the Te⋅I and the I⋅I distances in [**1⋅ChB**+2 Na+I]^+^ and [**1⋅XB**+2 Na+I]^+^, which are shorter than the corresponding distances in [**1⋅ChB**+I]^−^ and [1**⋅**XB+I]^−^ by about 0.1 Å (Table S3). From the molecular electrostatic potential (ESP) plots, the maximum ESPs at Te and I are 0.060 and 0.061 au, for **1⋅ChB** and **1⋅XB**, respectively (Figure 3). Upon sodium cation complexation, the maximum ESPs for [**1⋅ChB**+2 Na]^2+^ and [**1⋅XB**+2 Na]^2+^ become significantly more positive (0.188 and 0.152 au, respectively). That is the maximum ESPs in [**1⋅ChB**+2 Na]^2+^ and [**1⋅XB**+2 Na]^2+^ are higher than those in **1⋅ChB** and **1⋅XB**. These computational results allude to the source of the remarkable cooperativity of bromide and iodide halide binding exhibited by the sodium cation benzocrown ether complexed ChB and XB heteroditopic receptors. The cooperativity factor (*α*) for both **1⋅ChB** and **1⋅XB** can be rationalised from the increase in the calculated binding energies with halide, which is related to the increase in the magnitude of the bound cation induced polarisation.

**Table 2:** Relative free energies (in kcal mol^−1^) for the binding of iodide to the sigma hole type receptors **1⋅ChB** and **1⋅XB**.

Reaction^[a]^	Δ*G* [kcal mol^−1^]
**1⋅ChB** + I^−^ → [**1⋅ChB**+I]^−^	−4.65
[**1⋅ChB**+2 Na]^2+^ + I^−^ → [**1⋅ChB**+2 Na+I]^+^	−19.94
**1⋅XB** + I^−^ → [**1⋅XB**+I]^−^	−3.92
[**1⋅XB**+2 Na]^2+^ + I^−^ → [**1⋅XB**+2 Na+I]^+^	−22.23

[a] Optimisation and single‐point calculations were performed in CHCl_3_ solvent.

### Solid‐State Structure Determination

**Figure 4:**
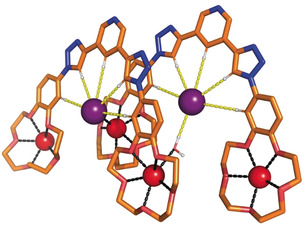
Solid‐state structure of the complex **1⋅HB⋅**2 NaI. For clarity non‐polar hydrogen atoms, selected counteranions and water molecules have been omitted, and only the higher occupancy components of the disordered regions are shown.

Further insight into the ion‐pair binding behaviour of the ditopic host systems was provided by solid‐state characterisation of the sodium iodide complex of the hydrogen‐bonding receptor **1⋅HB**. Crystals of the complex **1⋅HB⋅**2 NaI suitable for X‐ray structural determination were grown by slow evaporation of a chloroform:acetonitrile 9:1 solution of the receptor **1⋅HB⋅**2 NaPF_6_ containing two molar equivalents of tetrabutylammonium iodide (Figure 4). The cations are coordinated by the five oxygen atoms from the crown ether host, with Na^+^–O distances ranging from 2.302(10) Å to 2.477(10) Å and their coordination spheres are completed by additional interactions with water solvate molecules and iodide anions. The triazole‐bound iodide anion is encapsulated within each of the 3,5‐bis‐triazole pyridine anion‐binding clefts via an array of CH⋅⋅⋅I^−^ hydrogen bonding interactions.^29^, ^30^

## Conclusion

In conclusion, a series of novel heteroditopic receptors were synthesised, each containing benzo‐15‐crown‐5 ether cation binding groups covalently attached to 3,5‐bis‐iodine, tellurium‐ and proton‐functionalised‐triazole pyridine anion binding motifs providing respective XB, unprecedented ChB and HB ion‐pair receptors. ^1^H, ^13^C, ^125^Te and ^19^F NMR spectroscopy, ESI‐MS and X‐ray crystallography ion‐pair binding studies demonstrate the cooperative recognition of bromide and iodide halide anions via concomitant sodium cation crown ether complexation of the respective heteroditopic receptor. This can be rationalised by favourable electrostatics combined with the crown ether bound sodium cation facilitating an inductive withdrawal of electron density from the respective XB, ChB and HB anion recognition site. Importantly, determined cooperativity factors reveal the ChB heteroditopic receptor exhibits a remarkable and the largest enhancement of halide binding strength of over two hundred‐fold, in comparison to the XB and HB heteroditopic receptors. In effect, sodium cation–benzo‐crown ether binding by **1⋅ChB** switches on the recognition of bromide and iodide halide anions. DFT calculations suggest crown ether sodium cation complexation results in a polarisation of the ChB and XB heteroditopic receptor donors as a significant contribution to the origin of cooperativity exhibited by these systems. These observations provide evidence for a new mechanism of cooperativity for ion‐pair recognition unique to the mediation of sigma hole interactions via co‐bound cation recognition illustrating an exciting opportunity for their exploitation in future ion‐pair host design.

## Conflict of interest

The authors declare no conflict of interest.

## Supporting information

As a service to our authors and readers, this journal provides supporting information supplied by the authors. Such materials are peer reviewed and may be re‐organized for online delivery, but are not copy‐edited or typeset. Technical support issues arising from supporting information (other than missing files) should be addressed to the authors.

Supplementary
