# Supplementary material for: Chalcogen Bond Mediated Enhancement of Cooperative Ion‐Pair Recognition
Source: Angew Chem Int Ed Engl. 2020 May 12;59(29):12007–12. doi: 10.1002/anie.202001125 (PMC7383679; doi:10.1002/anie.202001125)
Supplement: Supplementary file 1 — Supplementary [file ANIE-59-12007-s001.pdf]

Angewandte Chemie
Eine Zeitschrift der Gesellschaft Deutscher Chemiker

Supporting Information

Chalcogen Bond Mediated Enhancement of Cooperative Ion-Pair Recognition

*Thanthapatra Bunchuay[+], Andrew Docker[+], Utt Eiamprasert, Panida Surawatanawong, Asha Brown, and Paul D. Beer**

anie_202001125_sm_miscellaneous_information.pdf

1. Solution binding studies……………………………………………………………S2
 1.1 Sodium cation complexation studies…………………………………………….S2
 1.2 ^{13}C NMR ion-Pair investigations for **1•XB**…………………………………S4
 1.3 ^{125}Te NMR Ion-Pair investigations for **1•ChB**…………………………….S5
 1.4 ^{1}H and ^{19}F NMR Ion-Pair investigations for **5**…………………………………S6
 1.5 Anion Binding studies……………………………………………………………S7
2. Synthetic Procedures and Characterisation……………………………………S9
3. Solid-Liquid extraction experiments………………………………………………...S25
4. Single crystal X-ray diffraction experiments……………………………………S29
5. DFT studies………………………………………………………………………S34

1. Solution binding studies

1.1 Sodium cation complexation studies

The sodium cation binding properties were studied by addition of two equivalents of $NaPF_6$ to ^1H-NMR solutions (10% d_6-DMSO-$CDCl_3$) of each host. In all cases significant downfield perturbations of the phenyl resonances as well as the crown ether ethylene protons were observed indicative of sodium cations binding in each crown ether group (Figure S1-3). Also perturbations of the respective pyridyl protons (H_a and H_b) were observed. To test whether the hexafluorophosphate anion was coordinating to the respective host's anion binding site, analogous titrations with two equivalents of $TBAPF_6$ were undertaken. No changes in the proton signals associated with the anion binding cleft were seen, confirming that the hexafluorophosphate anion does not participate in anion complexation.

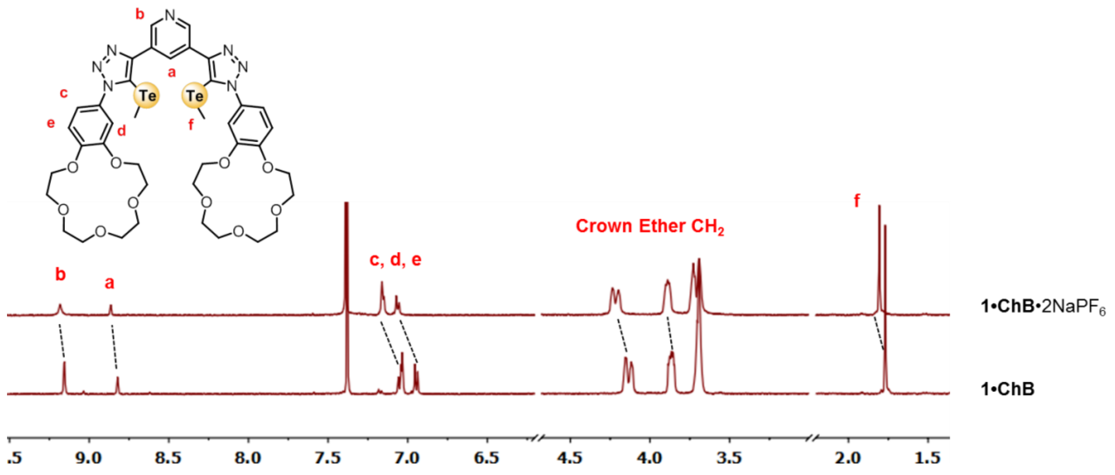

Figure S1. ^1H-NMR spectra of **1•ChB**, in the presence (top) and absence (bottom) of 2 equivalents of $NaPF_6$ in 10% d_6-DMSO/$CDCl_3$ (500MHz/ 298K).

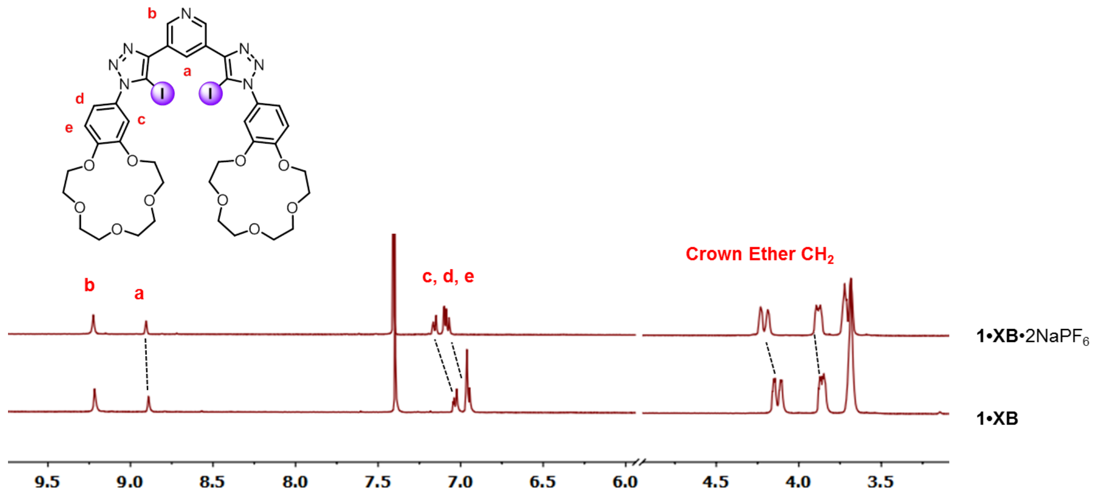

Figure S2. ^1H-NMR spectra of **1•XB**, in the presence (top) and absence (bottom) of 2 equivalents of $NaPF_6$ in 10% d_6-DMSO/$CDCl_3$ (500MHz/ 298K).

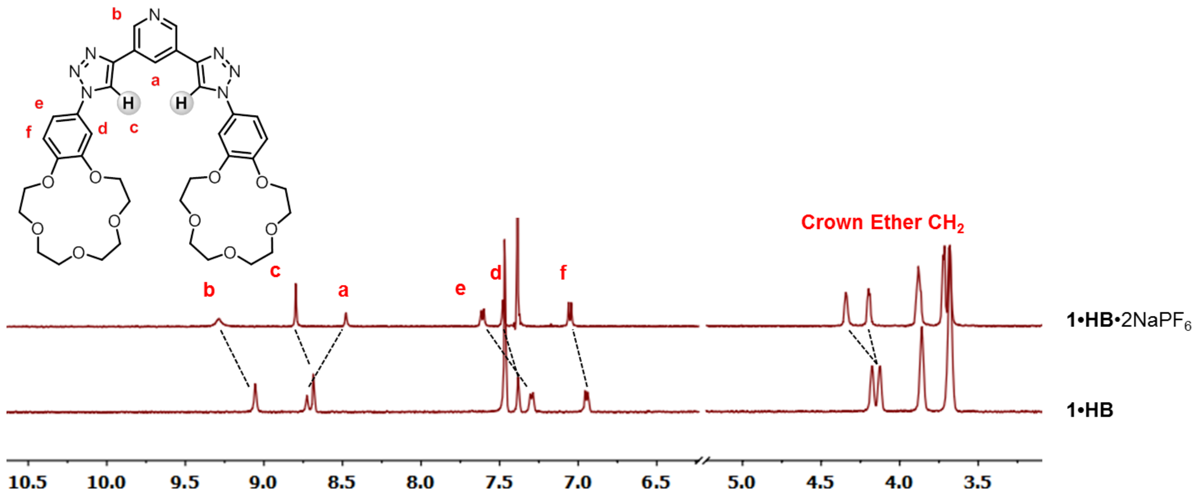

Figure S3. ^{1}H-NMR spectra of **1•HB**, in the presence (top) and absence (bottom) of 2 equivalents of $NaPF_6$ in 10% d_6-DMSO/$CDCl_3$ (500MHz/ 298K).

1.2 ^{13}C NMR Ion-Pair investigations for 1•XB

Further evidence for the participation of XB in the ion-pair recognition process was obtained by a comparison of ^{13}C-NMR spectra of **1•XB** in the presence of two equivalents of $NaPF_6$, and two equivalents of NaI. The chemical shift of the quaternary carbon of the bis-iodotriazole motif was significantly perturbed upfield with NaI (Δδ = -2.22 ppm). Iodide anion complexation was also confirmed by the upfield shift of internal pyridyl carbon (Δδ = -1.45 ppm) resulting from the halide anion being located in the bis-iodotriazole XB binding cleft of the receptor (Figure S4).

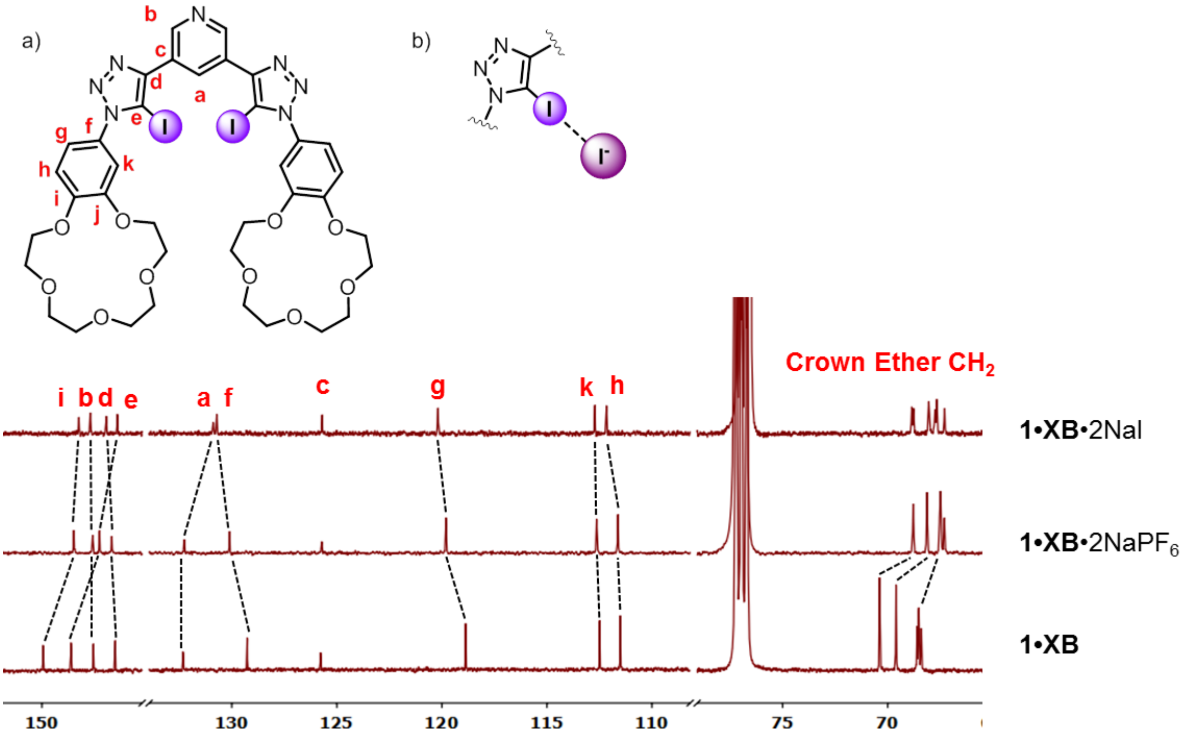

Figure S4. a) ^{13}C-NMR spectra of **1•XB**, with 2 equivalents of $NaPF_6$, with 2 equivalents of NaI in 10% d_6-DMSO in $CDCl_3$ (126 MHz, T = 298K) b) Proposed binding mode responsible for halogen bonding induced shift.

1.3 ^{125}Te NMR Ion-Pair investigations for 1•ChB

Analogous ^{125}Te-NMR experiments with **1•ChB** were carried out to provide evidence for chalcogen bond formation between the bis-methyltelluro triazole motif and iodide. Addition of two equivalents of $NaPF_6$ caused an upfield perturbation due to crown ether sodium cation complexation. By contrast, adding two equivalents of NaI induced a downfield shift of the ^{125}Te signal (Figure S5).

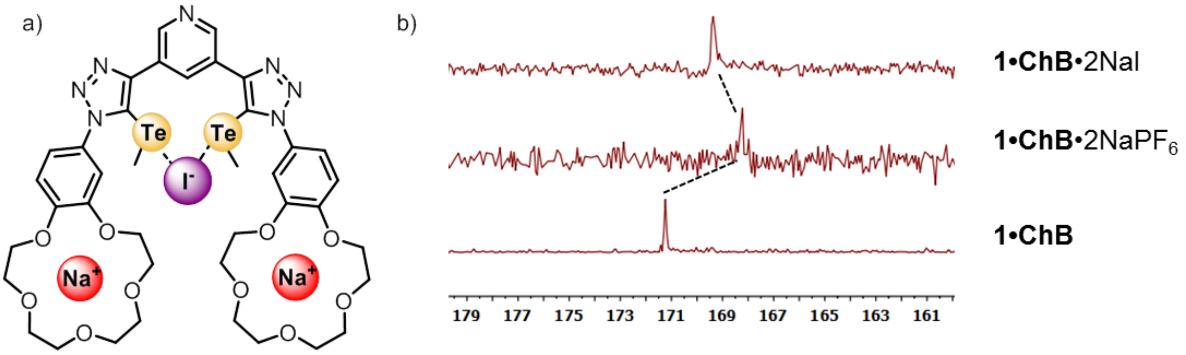

Figure S5. a) Proposed **1•ChB**•2NaI predominant complex b) ^{125}Te-NMR spectra of **1•ChB**, with 2 equivalents of $NaPF_6$, with 2 equivalents of NaI in 10% d_6-DMSO in $CDCl_3$ (158 MHz, T = 298K).

1.4 ^{1}H and ^{19}F NMR Ion-Pair investigations for 5

The fluorotriazole derivative serves as a non-halogen bond donor group. Qualitative ^{1}H-NMR spectroscopic experiments, revealed that the addition of 5 equivalents of TBAI to the bis-sodium cation crown ethers complexed fluorotriazole receptor **2.8** did not display any proton resonance perturbations indicating that no halide binding occurs (Figure S6b). Moreover, the ^{19}F-NMR spectra also showed no perturbation of the fluorine chemical environment of the fluoro-triazole groups on addition of iodide (Figure S6c).

a)
b)
c)

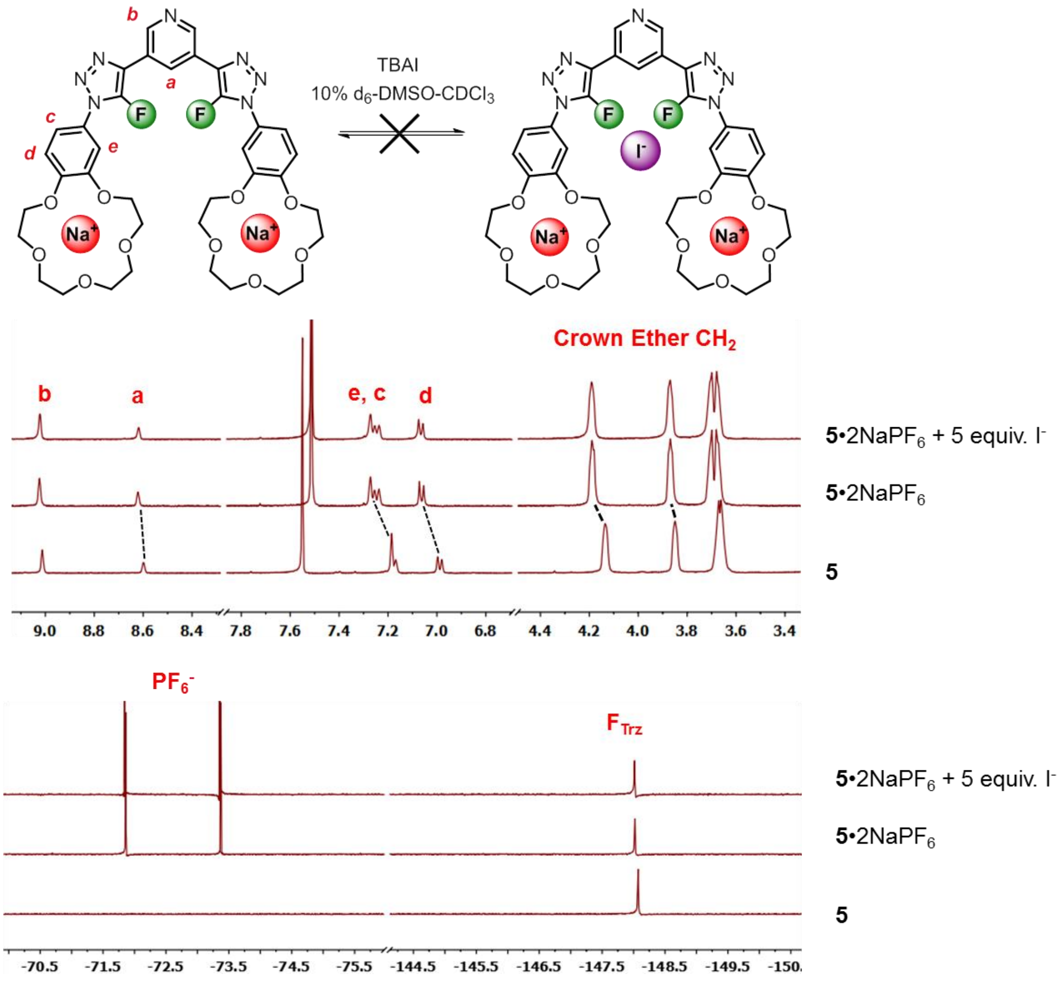

Figure S6. a) Postulated equilibrium of **5•**2NaPF6 in the presence of I$^-$ b) Truncated ^{1}H-NMR spectra and c) Truncated ^{19}F-NMR spectra of **5** in the presence of 2 equivalents of $NaPF_6$ and 5 equivalents of TBAI in 10% *d*$_6$-DMSO in $CDCl_3$ (500 MHz, T = 298K)**.**

1.5 Anion Binding studies

For sodium ion bound receptor titrations, the solution of 4mM $NaPF_6$ in 10% d_6-DMSO-$CDCl_3$ was used to dissolve solid host solution. Anion solution (100 mM) as the tetrabutylammonium salts were added in aliquots, the samples thoroughly shaken and spectra recorded. Spectra were recorded at 0, 0.2, 0.4, 0.6, 0.8, 1.0, 1.2, 1.4, 1.6, 1.8, 2.0, 2.5, 3.0, 4.0, 5.0, 7.0 and 10 equivalents. The anion binding isotherms in the presence and absence of 2 equivalents of $NaPF_6$ for the receptors **1•XB**, **1•ChB** and **1•HB** are shown in Figure S7-9.

In case of uncomplexed hosts titration, solid powder of host was dissolved in a selected solvent to obtain 2 mM of host. Anion solution (250 mM) as the the tetrabutylammonium salts were added in aliquots, the samples thoroughly shaken and spectra recorded.

In all cases where association constants were calculated, bound and unbound species were found to be in fast exchange on the NMR timescale. Stability constants were obtained by analysis of the resulting data using the WinEQNMR2328 software**.**

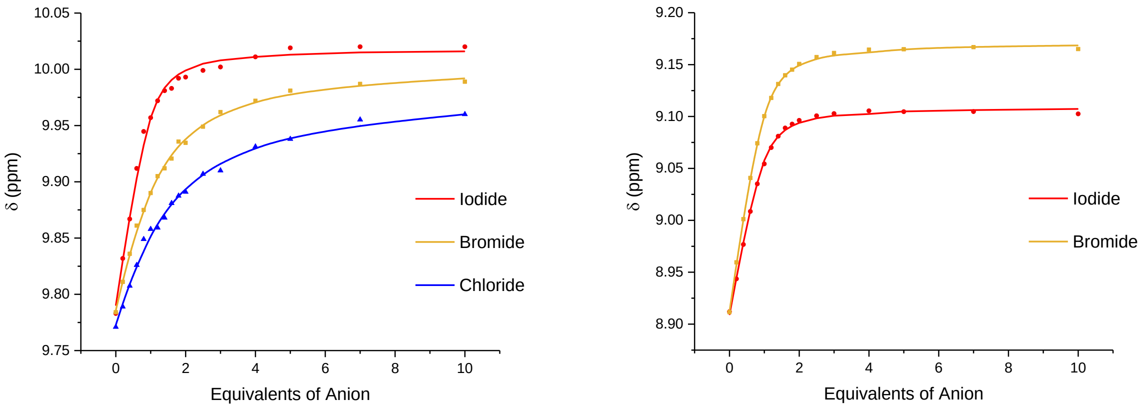

Figure S7. Anion Binding titration isotherms of **1•XB** (Left) and **1•XB**•2$NaPF_6$ (Right).

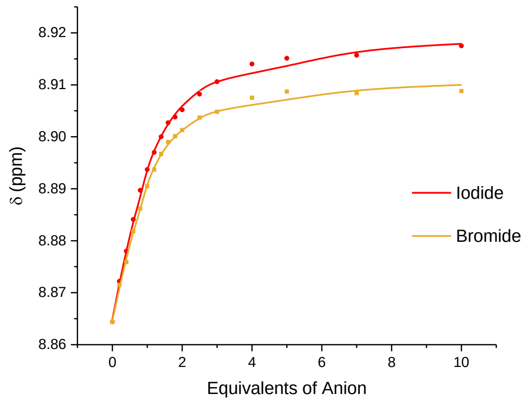

Figure S8. Anion Binding titration isotherms of **1•ChB**•2$NaPF_6$.

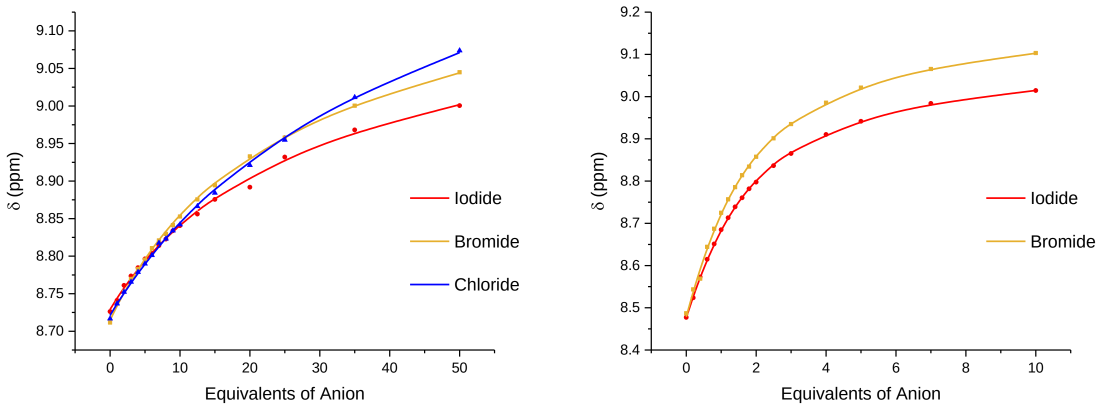

Figure S9. Anion Binding titration isotherms of **1•HB** (Left) and **1•HB**•$2NaPF_6$ (Right).

2. Synthetic Procedures and Characterisation

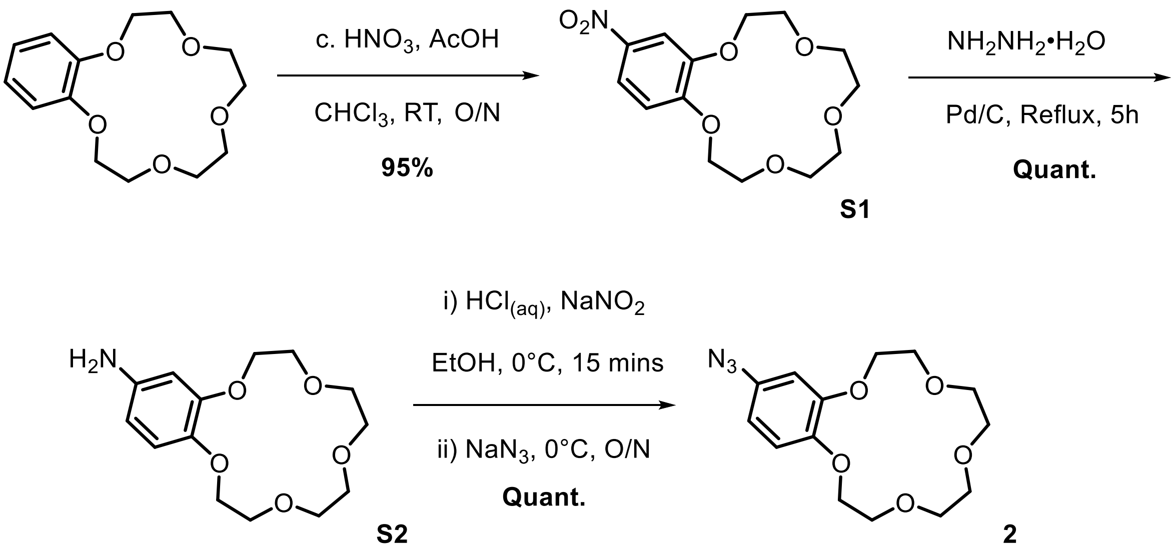

Figure S10. Synthesis of azido benzo-15-crown-5.

S1

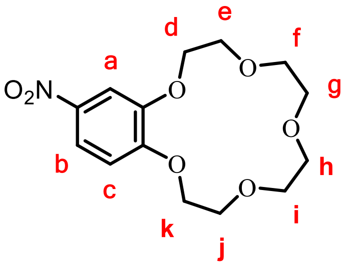

Benzo-15-crown-5 (1 g, 3.75 mmol) was dissolved in the mixture of $CHCl_3$ (22 ml) and acetic acid (17 ml) in round bottom flask, and cooled the mixture in an ice bath. Then, a cool solution of 70% HNO_3 (6.4 ml) in acetic acid (5ml) was slowly dropped to the mixture. The reaction was stirred overnight at room temperature. The organic phase was diluted with $CHCl_3$ and washed with water (50 ml), followed by 5% aqueous solution of Na_2CO_3 (50 ml). The organic residue was dried over $MgSO_4$ and removed under vacuum to get yellowish solid (95%).

^{1}H NMR (400MHz, CHLOROFORM-d) δ= 7.90 (1H, dd, J = 8.9 Hz, J = 2.6 Hz, H_b), 7.73 (1H, d, J = 2.6 Hz, H_b), 6.88 (1H, d, J = 8.9 Hz, H_c), 4.18 - 4.26 (4H, m, $H_{d,k}$), 3.91 - 3.98 (4H, m, $H_{e,j}$), 3.77 (8H, dquin, J = 3.4 Hz, J = 1.9 Hz, H_{f-i})

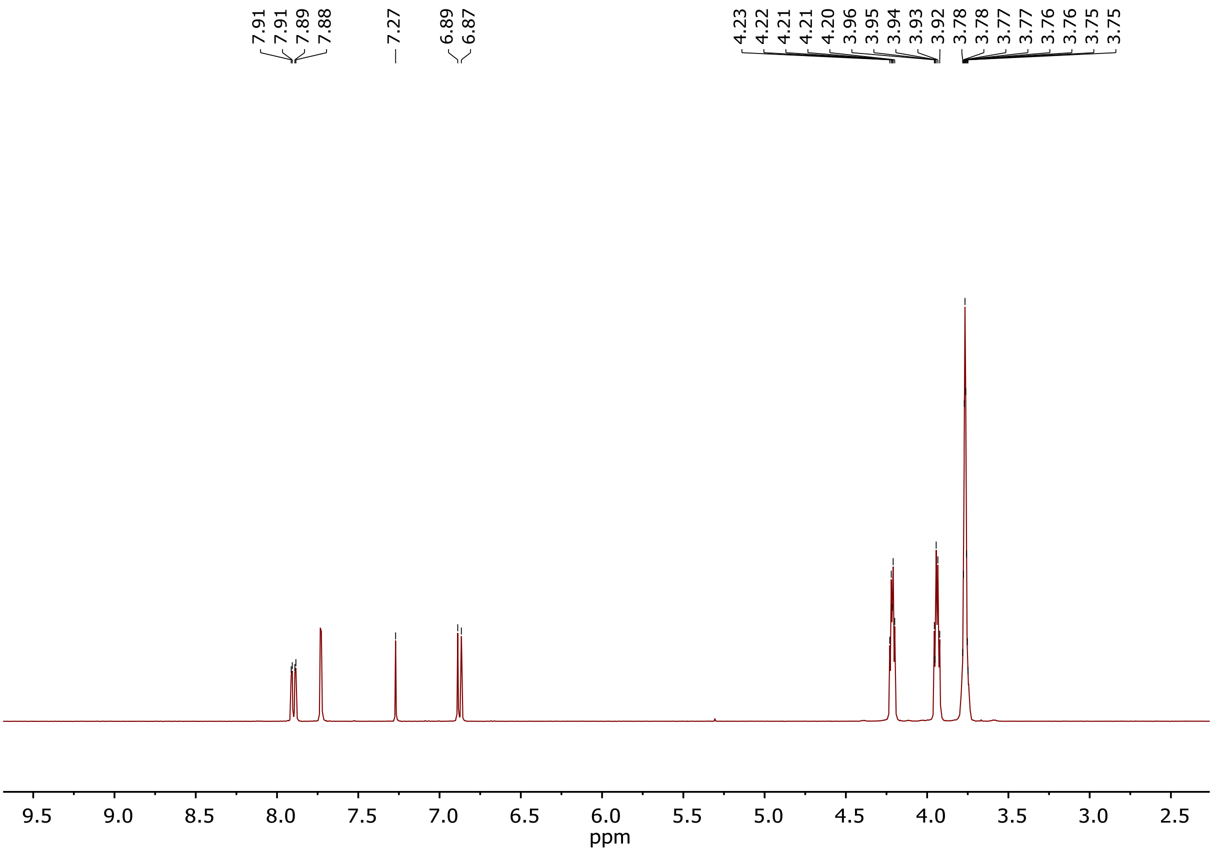

Figure S11. ^{1}H NMR spectrum of **S1**.

S2

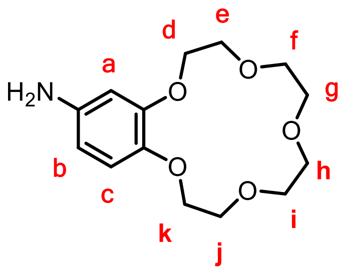

The mixture of Pd/C (10%loading) (123 mg) and hydrazine monohydrate (10 ml) were prepared in ethanol (22 ml), then NO_2-Benzo-15-crown-5 (1 g, 3.5 mmol) was added subsequently. The reaction was refluxed for 5h, and then solid residue was removed by filtering through Celite plug. The solvent was removed *in vacuo* obtaining an oil. The crude product was redissolved in DCM and dried over $MgSO_4$. Solvent was removed *in vacuo* to get colourless oil (Quant.).

^{1}**H NMR** (400MHz, CHLOROFORM-d) δ = 6.74 (1H, d, J = 8.4 Hz, H_c), 6.28 (1H, d, J = 2.6 Hz, H_a), 6.22 (1H, dd, J = 8.4 Hz, J = 2.6 Hz, H_b), 4.05 - 4.11 (4H, m, $H_{d,k}$), 3.85 - 3.92 (4H, m, $H_{e,j}$), 3.73 - 3.78 (8H, m, H_{f-i})

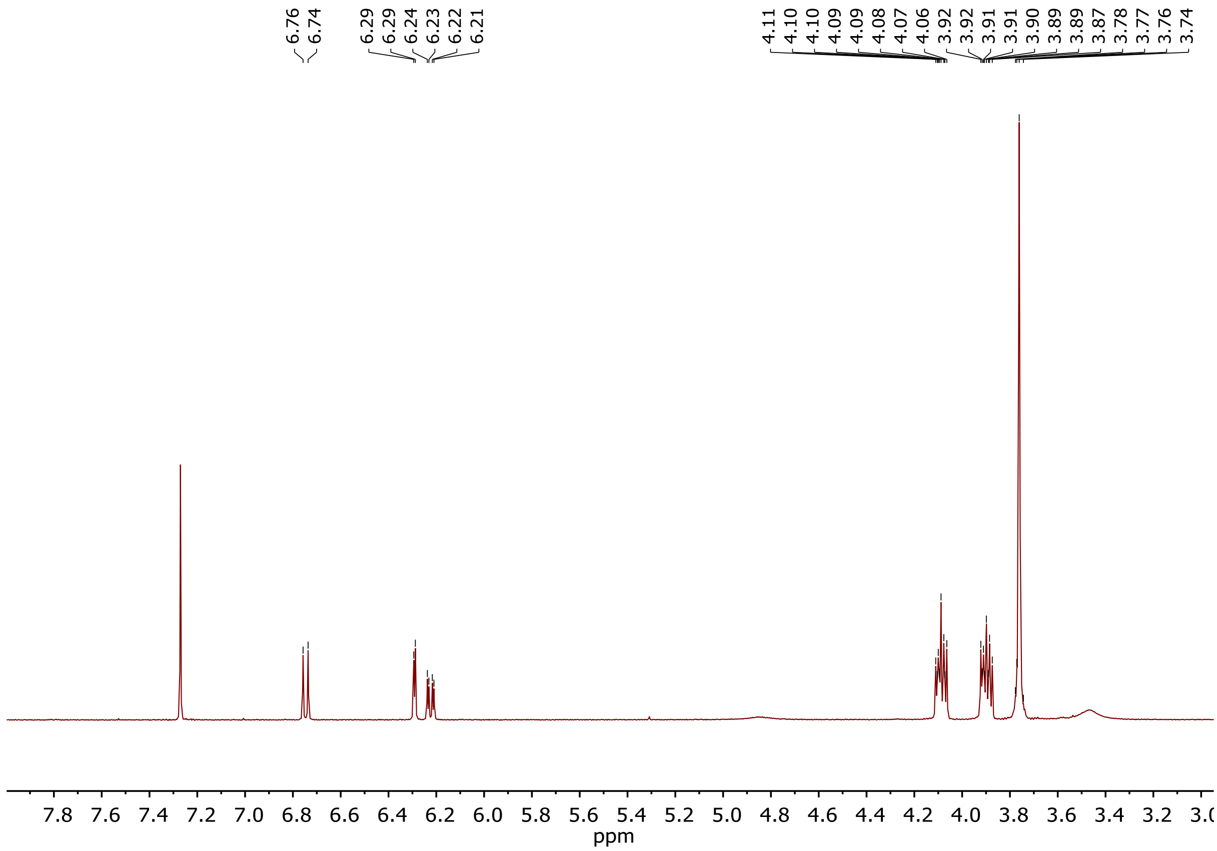

Figure S12. ^{1}H NMR spectrum of **S2**.

2

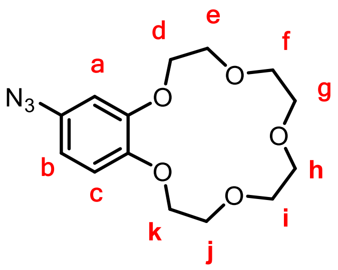

NH_2-Benzo-15-crown-5 (0.5 g, 1.76 mmol) was added to a solution of 37% HCl in water (1:1, 10 ml), followed by ethanol (5 ml). The reaction mixture was cooled to 0°C. $NaNO_2$ (0.18g, 2.5 mmol) was added portionwise with stirring further 15 minutes, then NaN_3 (0.16 g, 2.5 mmol) added while stirring at 0°C. The reaction mixture was allowed to room temperature and stirred overnight. The mixture was diluted with water and extracted with diethyl ether (3x30 ml). The combined organic phase was washed with $NaHCO_3$ (50 ml), brine (30 ml), dried over $MgSO_4$, and concentrated to dryness *in vacuo* to give the desired product as brown solid (Quant.). The compound was without further purification.

^{1}H NMR (400MHz, $CDCl_3$) δ = 6.86 (1H, d, J = 8.4 Hz, H_c), 6.59 (1H, dd, J = 8.6 Hz, J = 2.6 Hz, H_b), 6.53 (1H, d, J = 2.6 Hz, H_a), 4.10 - 4.15 (4H, m, $H_{d,k}$), 3.91 (4H, dt, J = 5.5 Hz, J = 3.5 Hz, $H_{e,j}$), 3.74 - 3.79 (8H, m, H_{f-i})

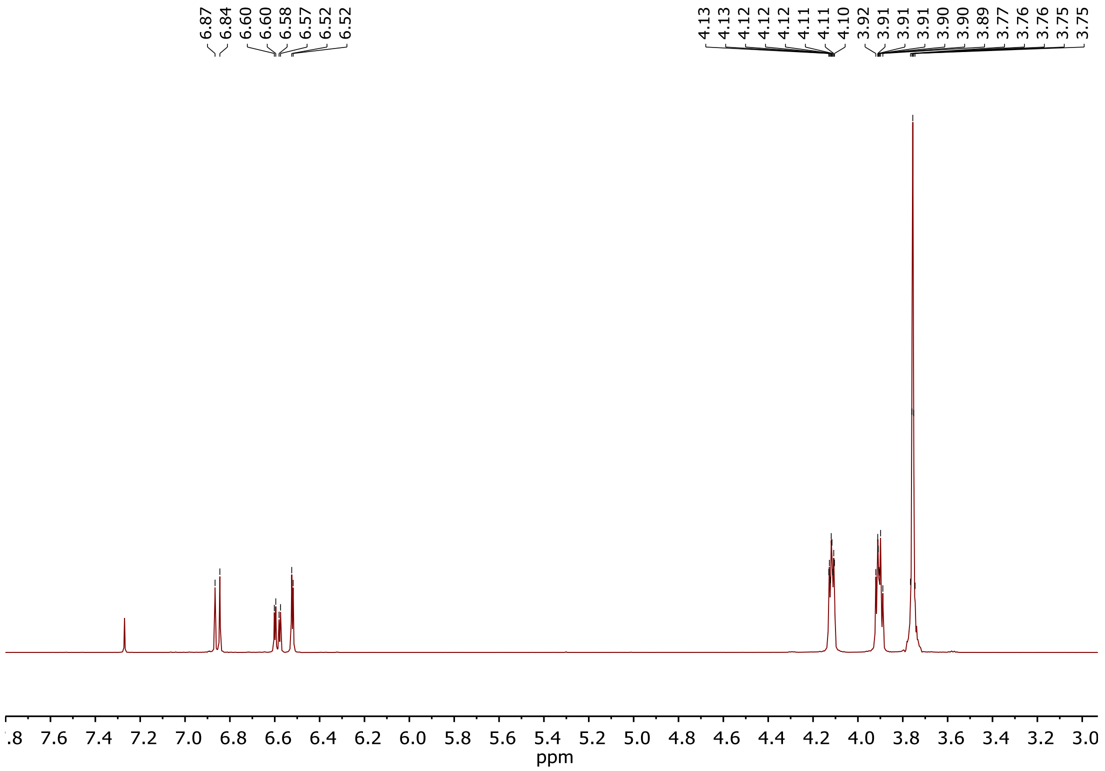

Figure S13. ^{1}H NMR spectrum of **2**.

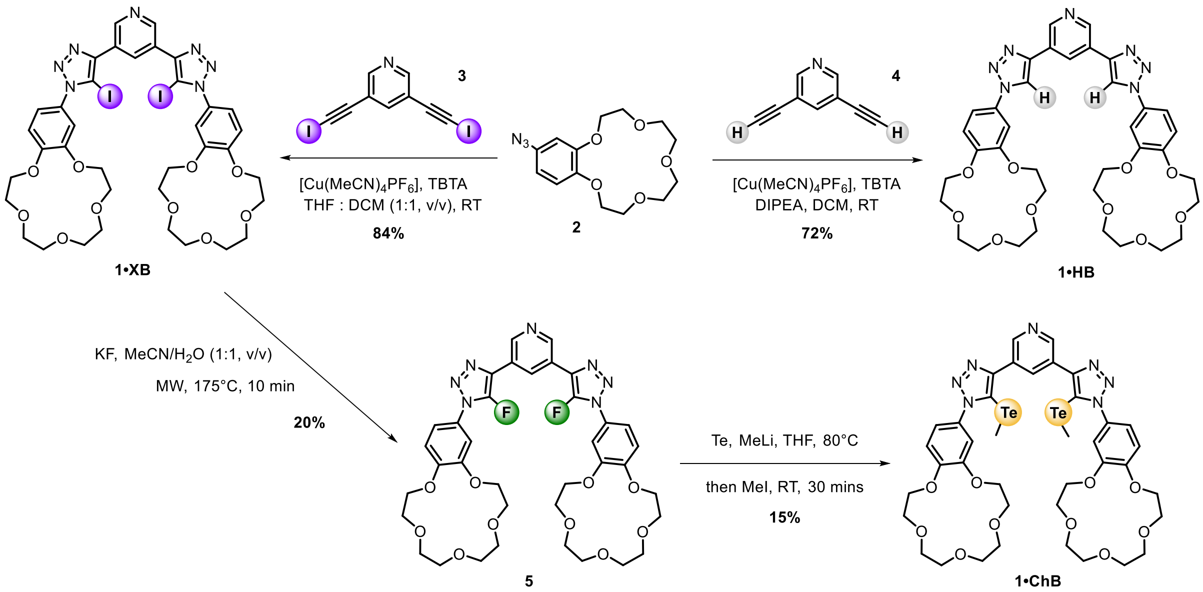

Figure S14. Synthesis of **1•ChB**, **1•XB** and **1•HB**.

.

1•XB

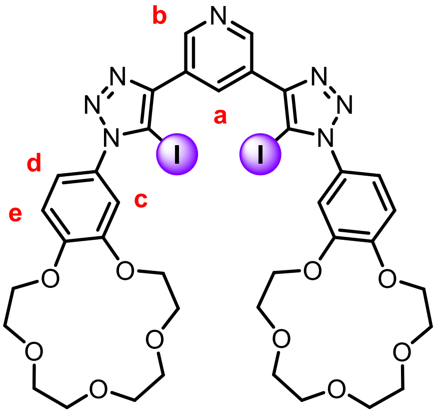

A solution of $[Cu(MeCN)_4]PF_6$ (34 mg, 0.09 mmol) and TBTA (48 mg, 0.09) in a mixture of 1:1 THF/DCM (3 ml) was added 3,5-diiodoethylnylpyridine **2.3** (114 mg, 0.3 mmol) and N_3-Benzo[15]crown-5 (204 mg, 0.66 mmol). The mixture was stirred under atmosphere of N_2 at room temperature for 3 days. Dichloromethane (10 mL) was added, and then the organic layer was washed with 35% aqueous ammonia solution (2 x 30 mL). The aqueous layer was extracted with dichloromethane (2 x20 mL) and the combined organics were wash with H_2O (3 x 30 ml). Solvent was removed *in vacuo* to obtain dark brown solid. The solid was washed with Et_2O (3 x30 ml); after which MeOH was added to the mixture, sonicated and decanted. The MeOH washing process was repeated for 3 times to get brown shiny solid (251 mg, 84%).

^{1}H-NMR (400 MHz, $CDCl_3$) δ = 9.27 (d, *J* = 2.2 Hz, 2H_b), 8.81 (t, *J* = 2.2 Hz, 1H_a) 7.07 – 7.00 (m, 2H_d), 6.99 – 6.91 (m, 4$H_{c,e}$), 4.15 (dt, *J* = 15.8, 4.3 Hz, 8H), 3.88 (dt, *J* = 9.0, 4.2 Hz, 8H), 3.76 – 3.70 (m, 8H), 3.71 (s, 16H)

^{13}C-NMR(126 MHz, $CDCl_3$) δ = 150.82, 149.47, 148.44, 147.36, 133.38, 129.93, 126.39, 119.59, 113.18, 112.21, 71.28, 70.50, 69.47, 69.38, 69.27, 69.20.

HRMS (ESI+ve) m/z: 998.10339 ($[M+H]^+$, $C_{37}H_{41}O_{10}N_7I_2$ requires 998.10770)

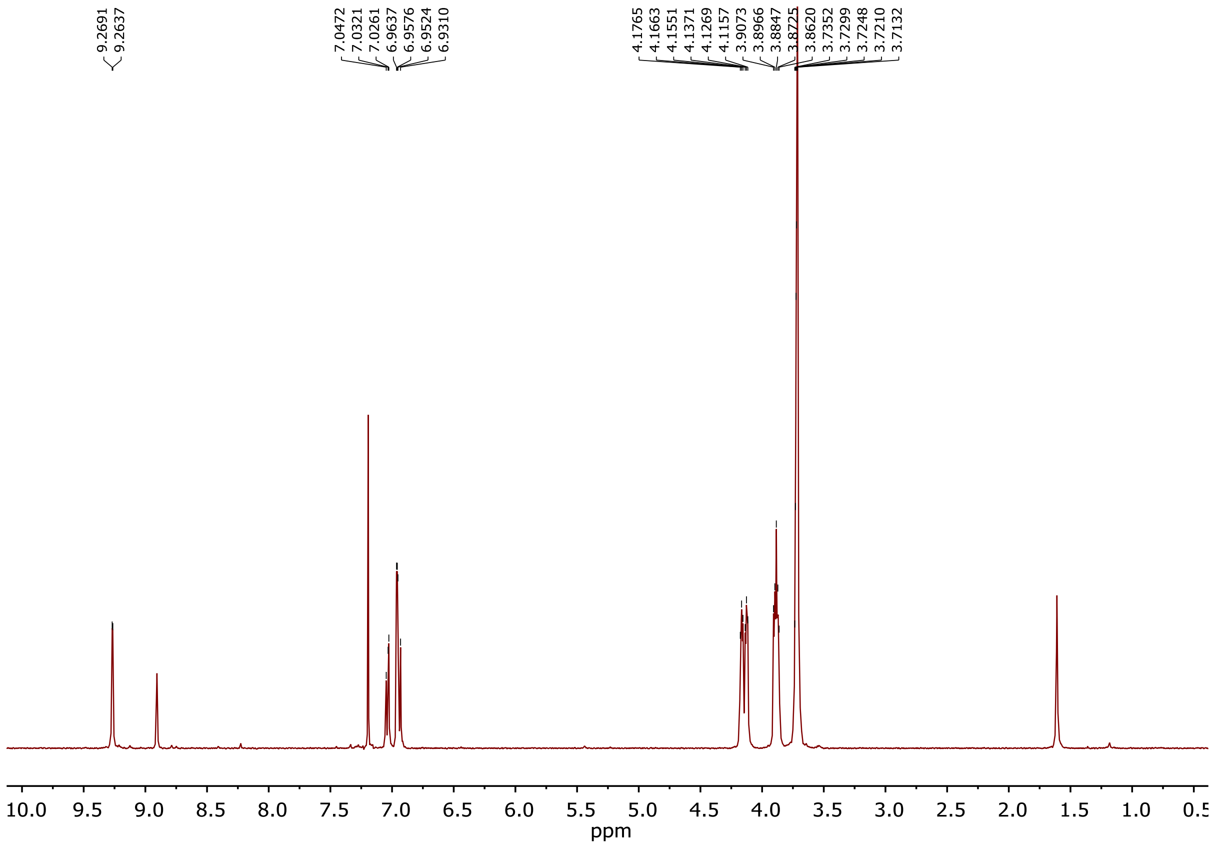

Figure S15. ^{1}H NMR spectrum of **1•XB**.

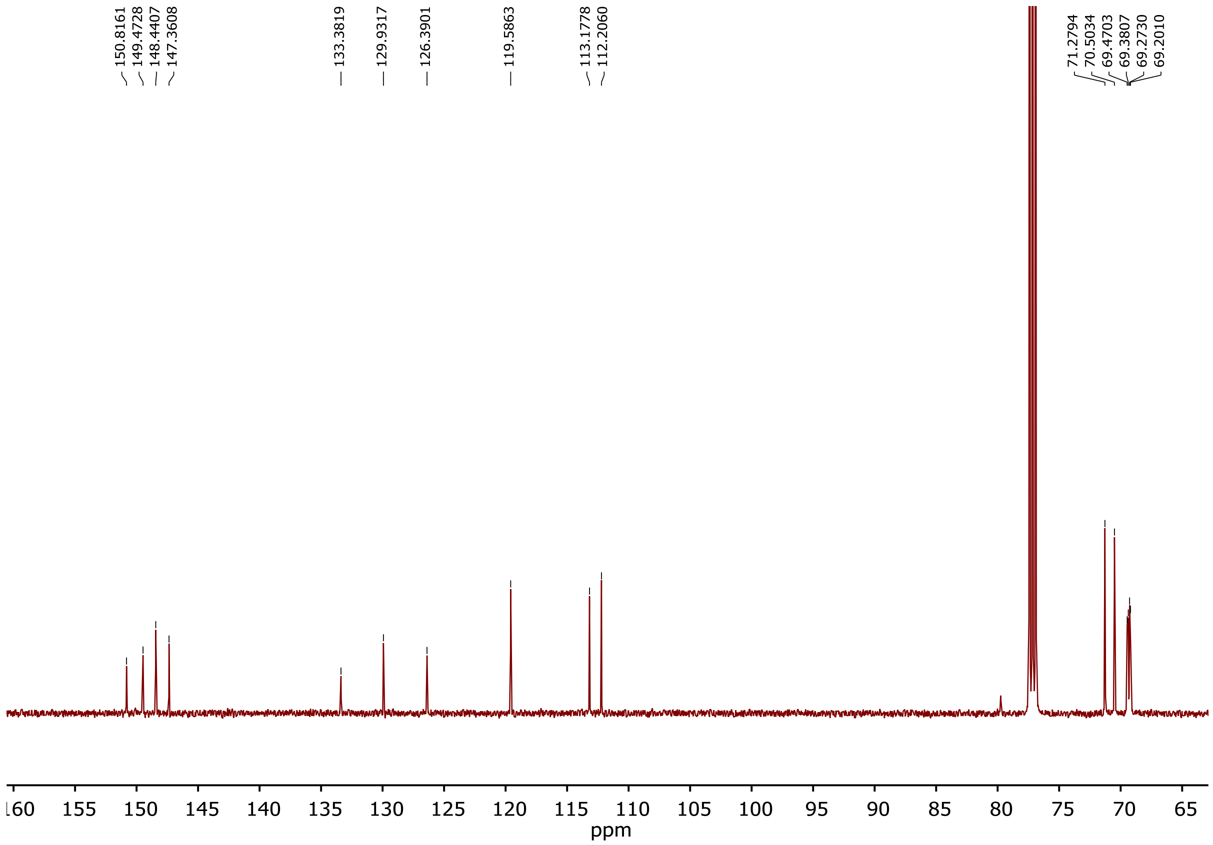

Figure S16. ^{13}C NMR spectrum of **1•XB**.

1•HB

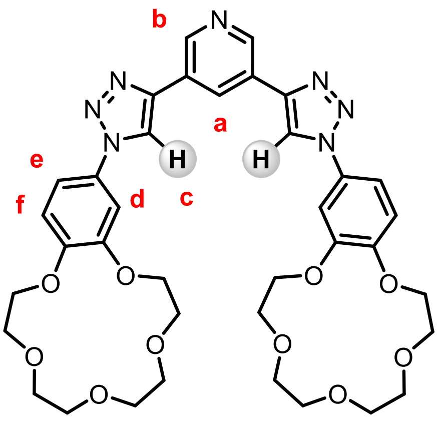

A solution of $[Cu(MeCN)_4]PF_6$ (16 mg, 0.045 mmol) and TBTA (23 mg, 0.045) in DCM (1 ml) was added 3,5-diethylnylpyridine (18.7 mg, 0.15 mmol), N_3-Benzo-15-crown-5 (100 mg, 0.323 mmol), and DIPEA (194 µl, 0.4 mmol). The reaction was stirred at room temperature for 2 days; after which the reaction was found to turn green. The product was purified with the same method for **1•XB**. The obtaining product is off white solid (109 mg, 72%)

^{1}H NMR (500 MHz, $CDCl_3$) δ = 9.12 (s, 2H_b), 8.75 (s, 1H_a), 8.31 (s, 2H_c), 7.40 (d, *J* = 2.5 Hz, 2H_d), 7.24 (m, 2H_e), 6.99 (d, *J* = 8.6 Hz, 2H_f), 4.29 – 4.23 (m, 4H), 4.23 – 4.18 (m, 4H), 3.96 (dt, *J* = 6.4, 3.3 Hz, 8H), 3.78 (m, 16H).

^{13}C NMR (126 MHz, $CDCl_3$) δ = 149.93, 149.72, 146.52, 144.86, 130.58, 129.87, 126.63, 118.71, 113.80, 112.95, 106.96, 71.09, 71.07, 70.40, 70.36, 69.38, 69.27, 69.20, 69.13.

HRMS (ESI+ve) m/z: 746.31421 ($[M+H]^+$, $C_{37}H_{44}O_{10}N_7$ requires 746.31442)

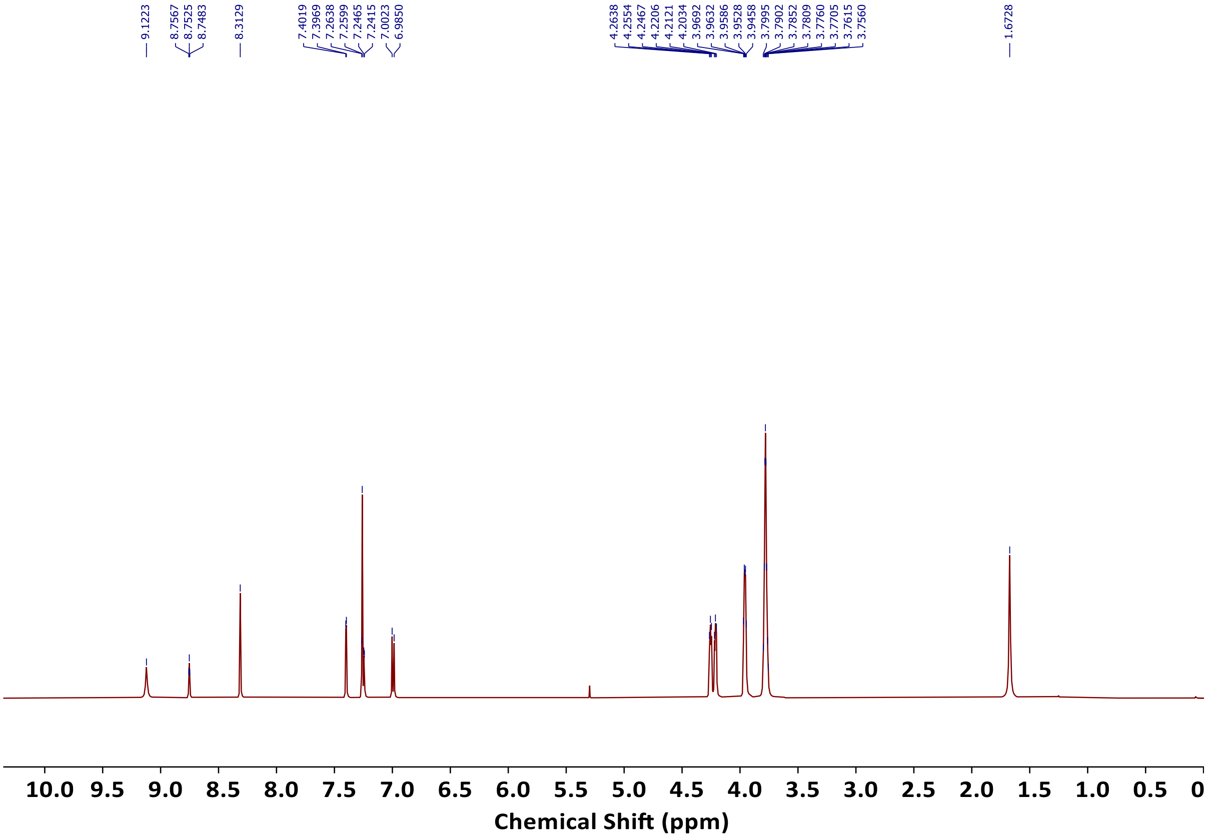

Figure S17. ^{1}H NMR spectrum of **1•HB**.

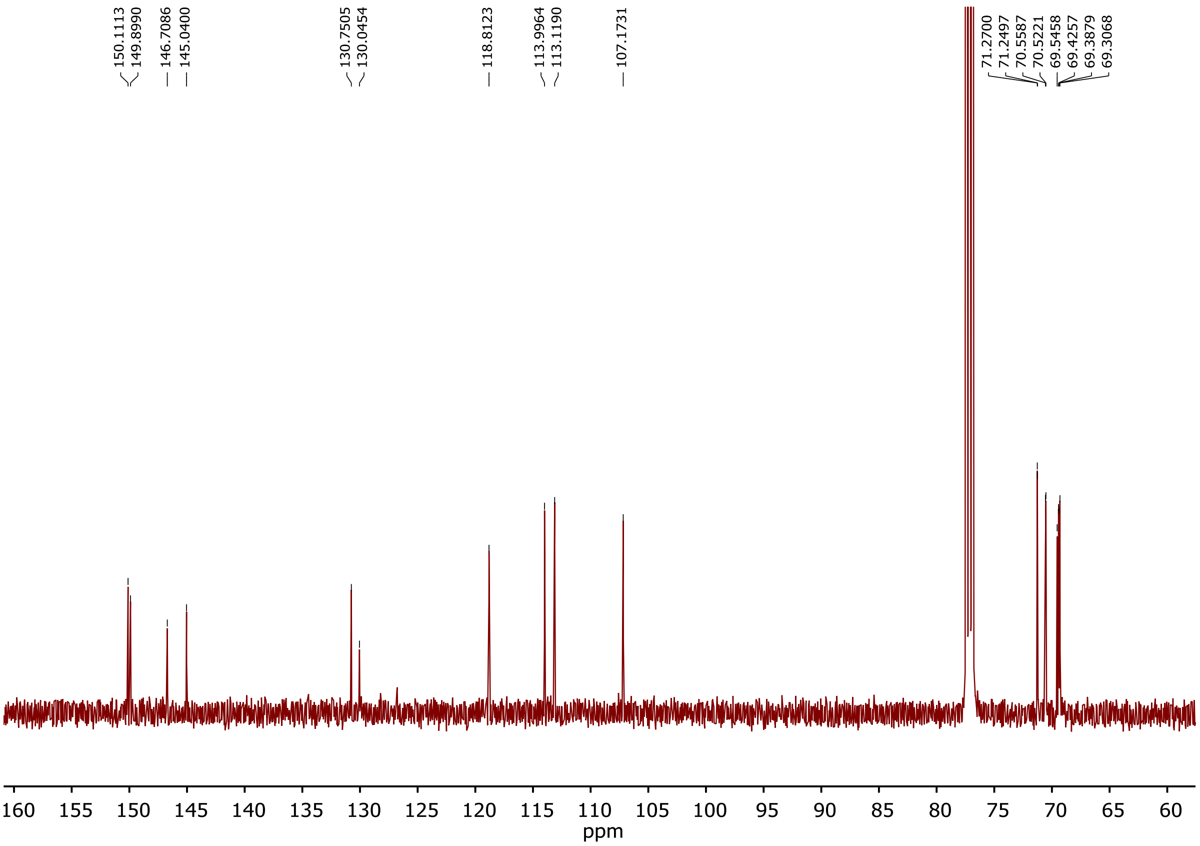

Figure S18. ^{13}C NMR spectrum of **1•HB**.

5

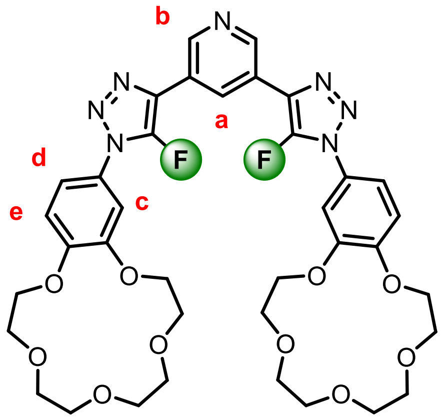

1•XB (200 mg, 0.200 mmol) was suspended in CH_3CN (5 ml) in a microwave vial, to which was added a solution of KF (120 mg, 2.07 mmol) in H_2O (5 ml), the vial was then sealed and heated by microwave irradiation to 175^0C for 10 minutes to obtain a brown solution. The reaction mixture was diluted with H_2O (50 ml) and $CHCl_3$ (50 ml) and organic and aqueous layers separated, the aqueous was then extracted by $CHCl_3$ (50 ml). The combined organics were then concentrated *in vacuo* to afford a brown residue, which was subjected to purification by preparatory thin layer chromatography using 7% MeOH/$CHCl_3$ as an eluent to afford **5** as an orange solid (31 mg, 20 %).

^{1}H-NMR (400 MHz, $CDCl_3$) δ = 9.15 (d, *J* = 2.0 Hz, 2H, H_b), 8.74 (s, 1H, H_a), 7.32 – 7.17 (m, 4H, under solvent peak, H_{c+d}), 7.03 (d, *J* = 8.6 Hz, 2H, H_e), 4.23 (dt, *J* = 6.8, 2.4 Hz, 8H, H), 3.96 (dq, *J* = 4.4, 1.9 Hz, 8H, H), 3.79 (dq, *J* = 3.8, 2.2 Hz, 16H).

^{13}C-NMR (101 MHz, $CDCl_3$) δ = 150.86, 150.36, 149.95, 148.01, 146.13, 146.09, 128.93, 127.19, 125.17, 125.12, 124.83, 124.75, 114.98, 114.94, 113.71, 108.39, 71.23, 70.50, 69.44, 69.35, 69.28, 69.25.

^{19}F-NMR (376 MHz, $CDCl_3$) δ = -148.01.

HRMS (ESI+ve) m/z: 782.29547 ($[M+H]^+$, $C_{37}H_{42}O_{10}N_7F_2$ requires 782.29557)

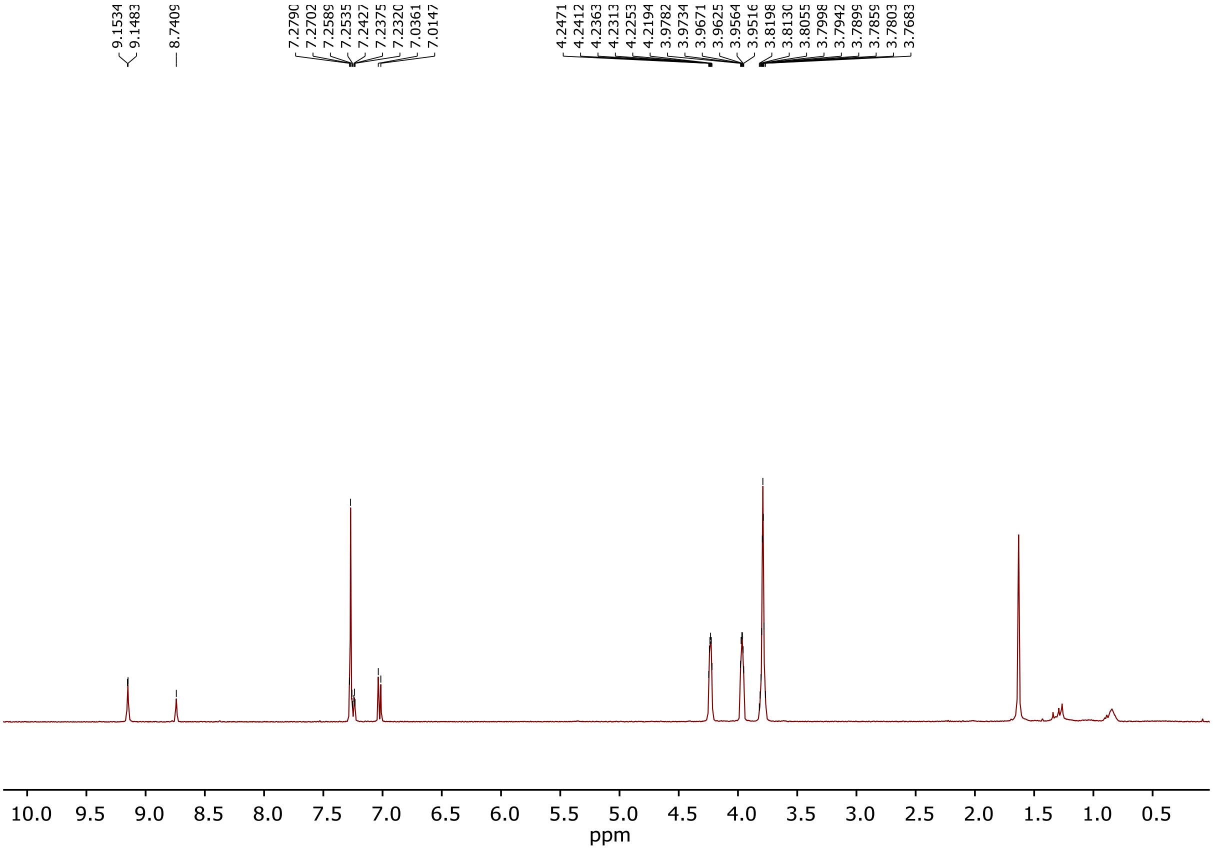

Figure S19. ^{1}H NMR spectrum of **5**.

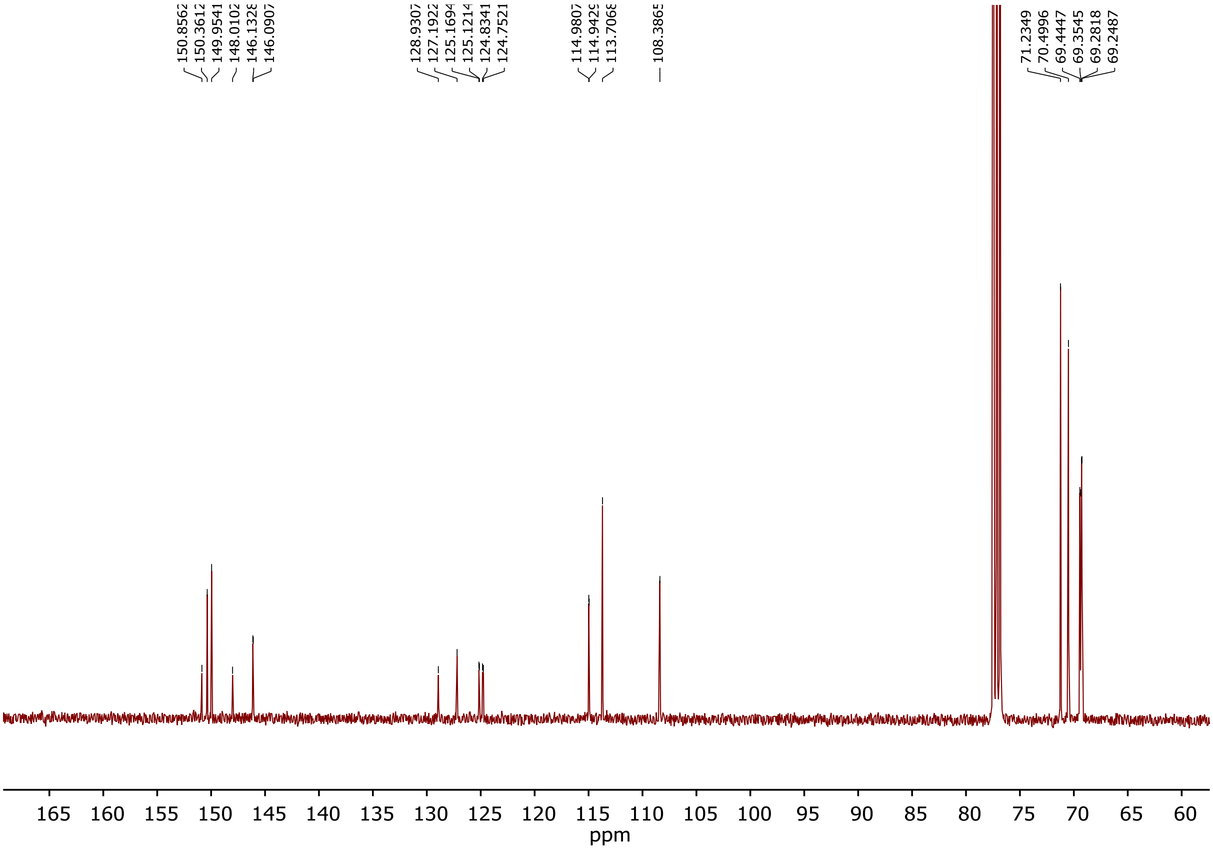

Figure S20. ^{13}C NMR spectrum of **5**.

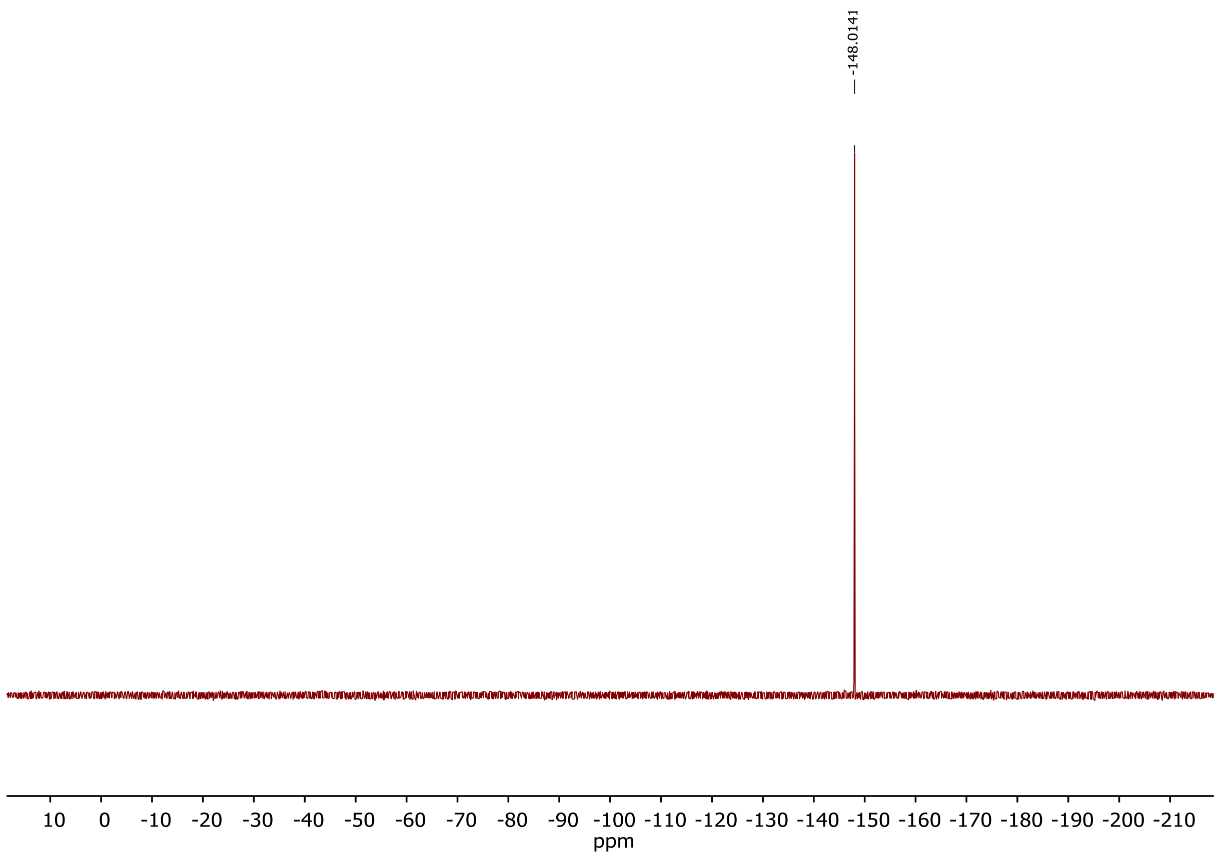

Figure S21. ^{19}F NMR spectrum of **5**.

1•ChB

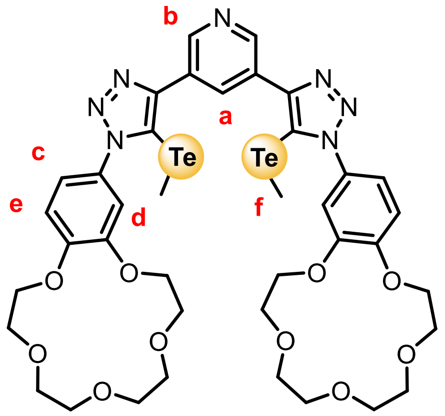

Tellurim powder (65 mg, 0.509 mmol) suspended in a minimum of dry THF and the sealed vial cooled to 0^0C, to which was added a solution of a 1.6M solution of MeLi in diethyl ether (0.27 ml, 0.432 mmol). After the suspension had warmed to room temperature the mixture was sonicated until the formation of a brown suspension was evident and left to stir for 1 hour. Separately a suspension of **5** (59 mg, 0.0755 mmol) in a minimum of THF was made and added dropwise to the brown suspension, the mixture was then heated in the dark at 80^0C for 4 hours. The mixture was then allowed to warm to room temperature and MeI (18.3 µl, 0.294 mmol) added and left to stir for 30 minutes. After which time the mixture was diluted with H_2O (50 ml) and extracted into $CHCl_3$ (50 ml), collected organic phase was dried over MgSO4 and subjected to purification by preparatory thin layer chromatography (eluent: 7 % MeOH/$CHCl_3$) to afford the **1•ChB** as an orange-brown solid (11.7 mg, 0.0113 mmol, 15 %).

^{1}**H NMR** (500 MHz, Chloroform-*d*) δ = 9.27 (s, $2H_b$), 8.92 (s, $1H_a$), 7.13 (dd, *J* = 8.4, 2.4 Hz, $2H_c$), 7.11 (d, *J* = 2.4 Hz, $2H_d$), 7.01 (d, *J* = 8.4 Hz, $2H_e$), 4.23 (dt, *J* = 17.6, 4.1 Hz, 8H), 3.97 (dt, *J* = 11.2, 4.4 Hz, 8H), 3.80 (th, *J* = 6.2, 3.3 Hz, 16H), 1.85 (s, $6H_f$).

^{13}**C NMR** (126 MHz, $CDCl_3$) δ = 150.66, 150.45, 149.39, 148.80, 134.80, 131.09, 127.44, 119.17, 113.12, 112.02, 101.21, 71.24, 70.48, 69.48, 69.38, 69.21, 69.15, -12.88.

^{125}**Te NMR** (158 MHz, $CDCl_3$) δ =166.77.

HRMS (ESI+ve) m/z: 1030.15585 ($[M+H]^+$, $C_{39}H_{48}N_7O_{10}Te_2$ requires 1032.15460)

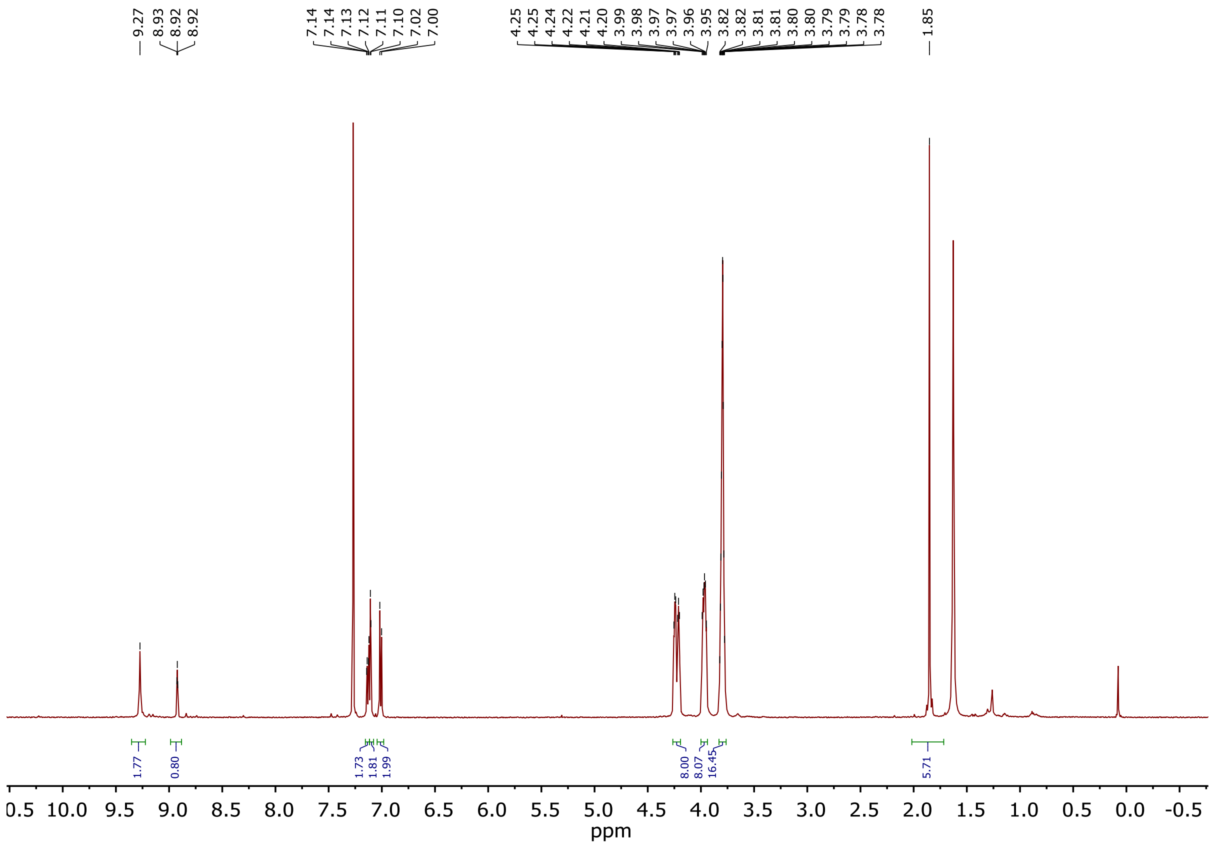

Figure S22. ^{1}H NMR spectrum of **1•ChB**.

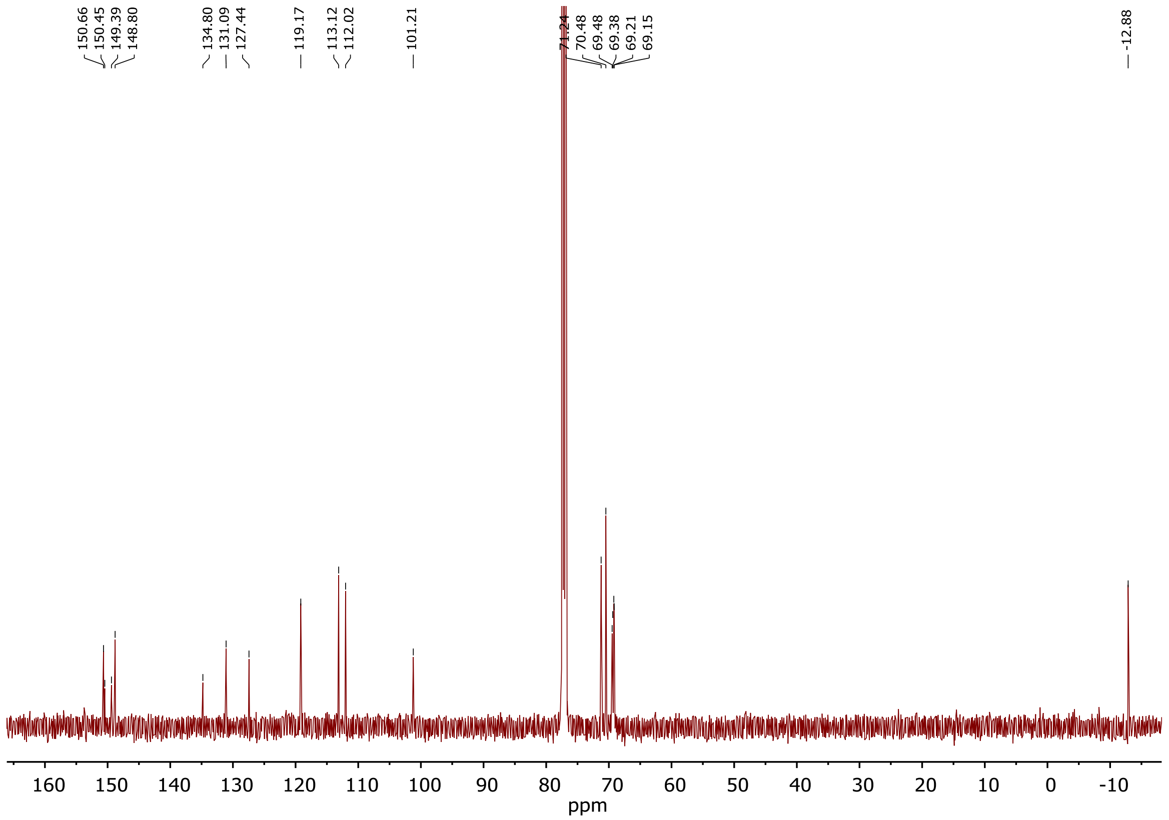

Figure S23. ^{13}C NMR spectrum of **1•ChB**.

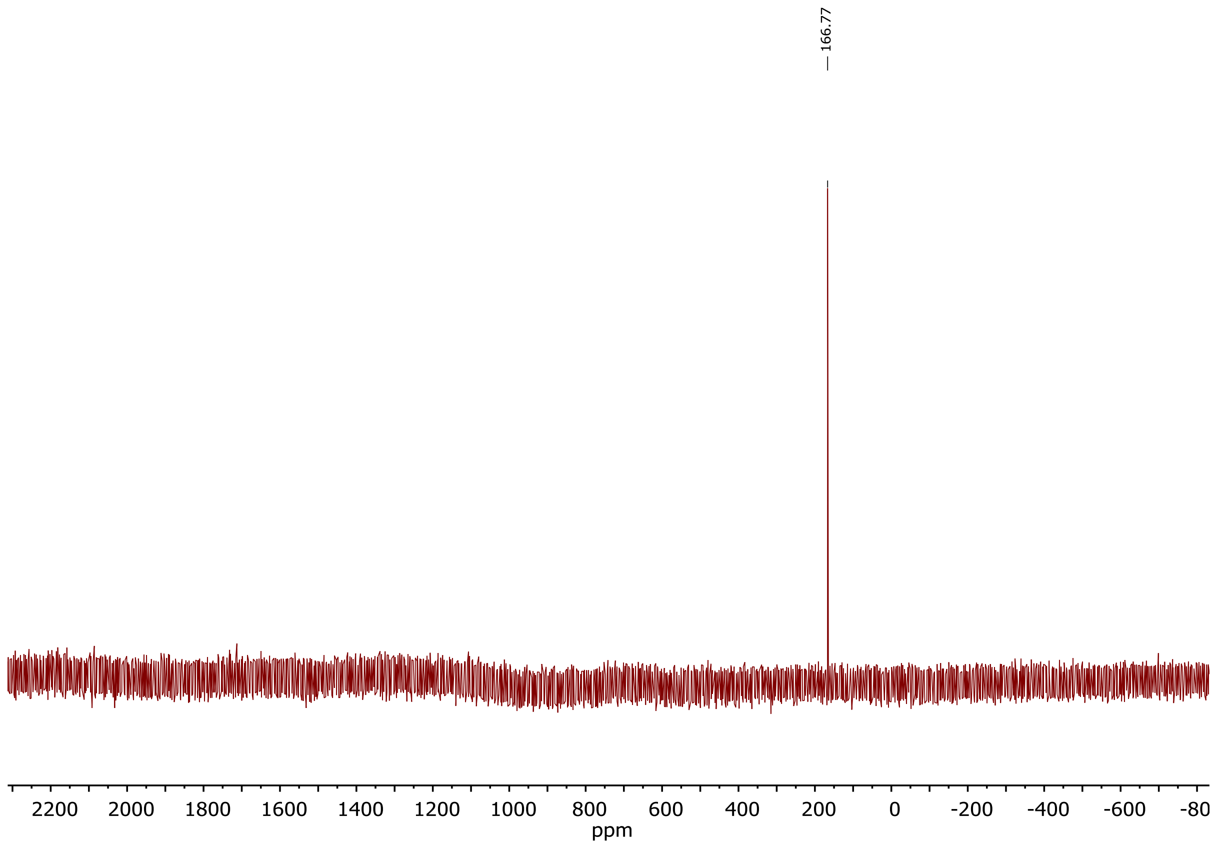

Figure S24. ^{125}Te NMR spectrum of **1•ChB**.

3. Solid-Liquid extraction experiments

The ability of ion-pair receptors to extract and solubilise salts into organic media is another interesting application of the ditopic host. While a number of alkali metal halide salt HB based systems are known. To the best of our knowledge, exploiting sigma-hole type ion pair receptors in the study of solid-liquid salt extraction is unprecedented.

Solid-liquid extraction studies were carried out by exposing a selected organic solvent solution of each ditopic receptor to an excess of microcrystalline sodium halide salts. After 1 h of sonication at ambient temperature, the solution was filtered and analysed by ^{1}H-NMR spectroscopy and electrospray mass spectrometry (ESI-MS). Analogous control experiments were conducted to confirm the insolubility of the salt ion the solvent mixture 10% $CD_3CN/CDCl_3$ in an analogous fashion as previously reported.[1]

The organic solvent mixture of 10% $CD_3CN/CDCl_3$ was found to be the most suitable for the extraction studies with NaX (X = Cl, Br, I, NO_3) salts. Significant ^{1}H-NMR chemical shift changes were observed for **1•XB** with $NaNO_3$, NaBr and NaI whilst the signals from the NaCl extraction remained unchanged (Figure S25). The downfield perturbations of the receptor's internal pyridyl proton H_a observed in NaBr and NaI extraction experiments indicated that extraction had occured. High resolution ESI-MS revealed signals corresponding to [**1•XB** +2Na+X]$^{+}$ where X is Br^- and I^- (Figure S26). With **1•HB**, NaBr and NaI extraction solutions revealed the significant ^{1}H-NMR chemical shift changes, whilst no perturbation of proton resonances were noted with NaCl and $NaNO_3$ (Figure S27). The ESI-MS analysis showed signals corresponding to [**1•HB** +2Na^{+}+X^{-}]$^{+}$ where X is Br^- and I^- (Figure S28). Successful solid-liquid extraction of NaBr and NaI was also seen by ^{1}H-NMR spectra of **1•ChB** (Figure S29), however ESI-MS evidence displayed only signals corresponding to [**1•ChB** +X]$^{-}$ where X = Br and I (Figure S30).

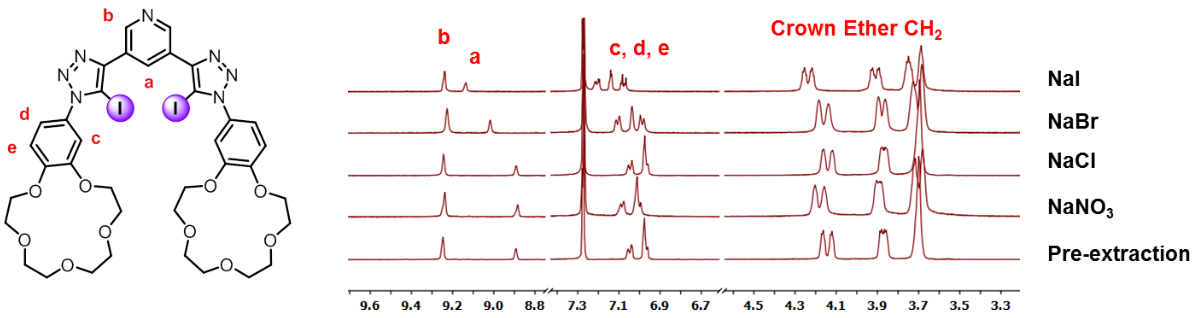

Figure S25 ^{1}H-NMR spectrum of (a) pre- and post- solid/liquid extraction experiments of **1•XB** receptor with $NaNO_3$, NaCl, NaBr or NaI in 10%$CD_3CN/CDCl_3$.

a)

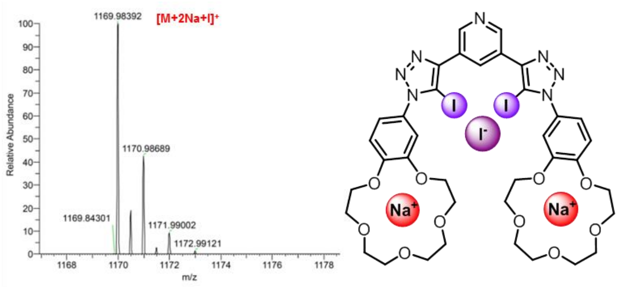

b)

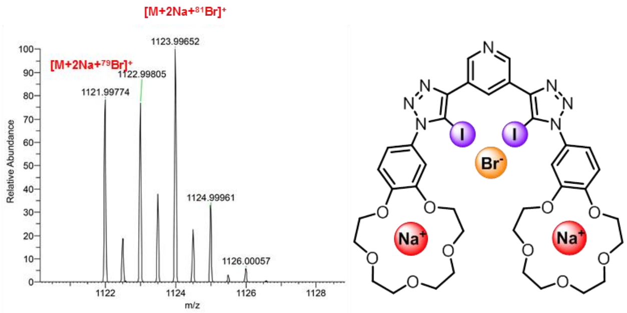

Figure S26 ESI-MS of post-extraction solution of sodium complexed **1•XB** with a) NaI and b) NaBr in 10%MeCN/$CHCl_3$ at 298K; where M is m/z of **1•XB**.

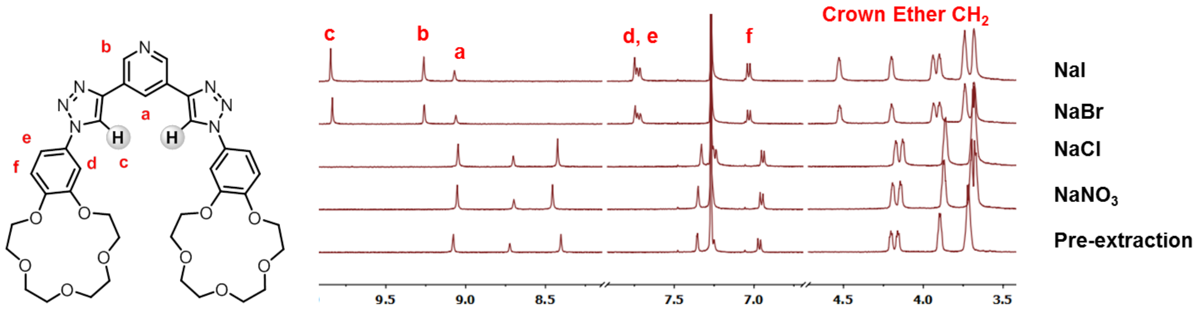

Figure S27 ^{1}H-NMR spectrum of pre- and post- solid/liquid extraction experiments of **1•HB** receptor with $NaNO_3$, NaCl, NaBr or NaI in 10%$CD_3CN/CDCl_3$.

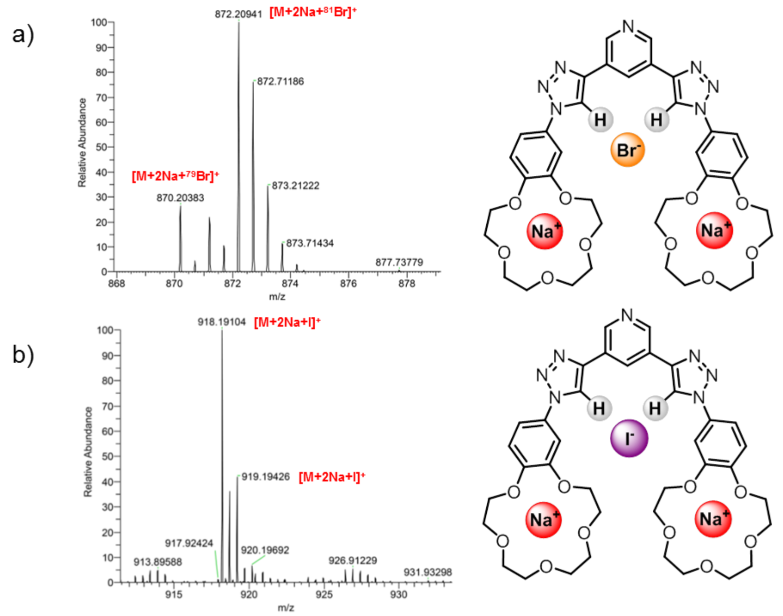

Figure S28 ESI-MS of post-extraction solution of sodium complexed **1•HB** with a) NaBr and b) NaI in 10%MeCN/$CHCl_3$ at 298K; where M is m/z of **1•HB**.

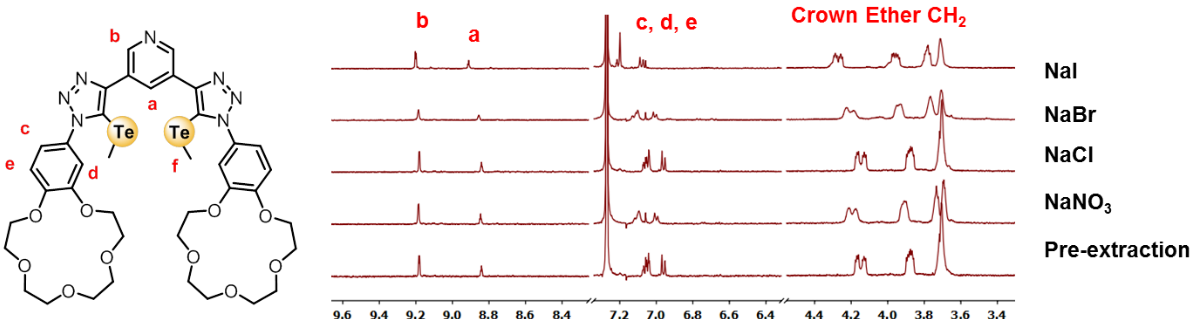

Figure S29 ^{1}H-NMR spectrum of pre- and post- solid/liquid extraction experiments of **1•ChB** receptor with $NaNO_3$, NaCl, NaBr or NaI in 10%CD_3CN/$CDCl_3$.

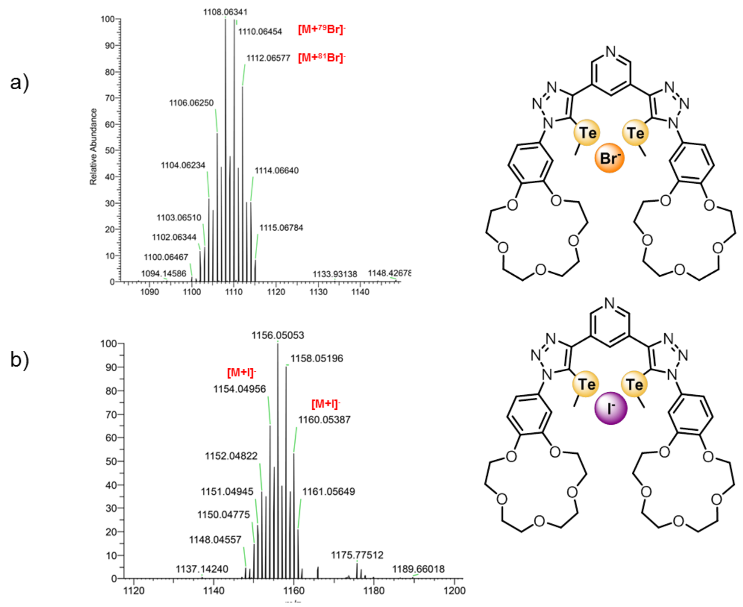

Figure S30 ESI-MS of post-extraction solution of sodium complexed **1•ChB** with a) NaBr and b) NaI in 10%MeCN/$CHCl_3$ at 298K; where M is m/z of **1•ChB**.

References for Section 3

[1] B. Akhuli, P. Ghosh, *Chem. Commun.* **2015**, *51*, 16514–16517

4. Single crystal X-ray diffraction experiments

Data collection

Diffraction data for the structures of the complexes **1•HB**•2NaI and **1•XB**•HCl were collected at 100 K using silicon double crystal monochromated synchrotron radiation (λ = 0.6889 Å) at Diamond Light Source, beamline I19,[S1] using a custom-built Crystal Logic diffractometer. Unit cell parameter determination and refinement and raw frame data integration were carried out using the CrysAlisPro[S2] package.

Structures were solved by charge-flipping methods using SUPERFLIP[S3] and refined by full matrix least squares on F^2 using the CRYSTALS suite.[S4] All non-hydrogen atoms were refined with anisotropic displacement parameters. A more detailed discussion of each individual structure is given below, including a description of hydrogen atom treatment for each structure.

Sodium iodide ion-pair complex of the hydrogen-bonding receptor 1•HB•2NaI

Crystals of the complex **1•HB**•2NaI suitable for X-ray structural determination were grown by slow evaporation of a chloroform:acetonitrile 9:1 solution of the receptor 1•HB•2NaPF$_6$ containing two molar equivalents of tetrabutylammonium iodide. The crystals were small and weakly diffracting; despite the use of synchrotoron radiation, the data are of relatively low quality. Consequently, where necessary, geometric restraints to bond lengths and angles were applied to ensure a physically reasonable model and thermal and vibrational restraints were applied to maintain sensible anisotropic displacement parameters. Some positional disorder was identified within the molecular framework of the ditopic ligand **1•HB**. To account for this three of the four triazole groups were modelled over two positions using refined partial occupancies. Comparatively large anisotropic displacement ellipsoids for two of the four benzotriazole groups were also observed, which may indicate further positional disorder within these regions of the structure. Since the quality of the data is insufficient to permit sensible modelling of these regions using two discrete positions for each atom, tight thermal restraints were instead applied. The two iodide counteranions which are not bound within the 3,5-bis-triazole pyridine anion recognition units were modelled over five partially occupied positions. Several partially occupied water molecules are also present within the asymmetric unit. Hydrogen atoms were generally visible in the Fourier difference map. Those attached to carbon atoms were initially positioned geometrically and refined against the data with restraints on bond lengths and angles, after which their positions were used as the basis for a riding model.[S5] Most of the hydrogen atoms on the water molecules were not clearly visible in the difference maps and it was not possible to sensibly refine their positions. These hydrogen atoms were inserted at idealised hydrogen bonding positions with O—H distances of 0.9 Å and constrained to ride on the attached oxygen atoms.

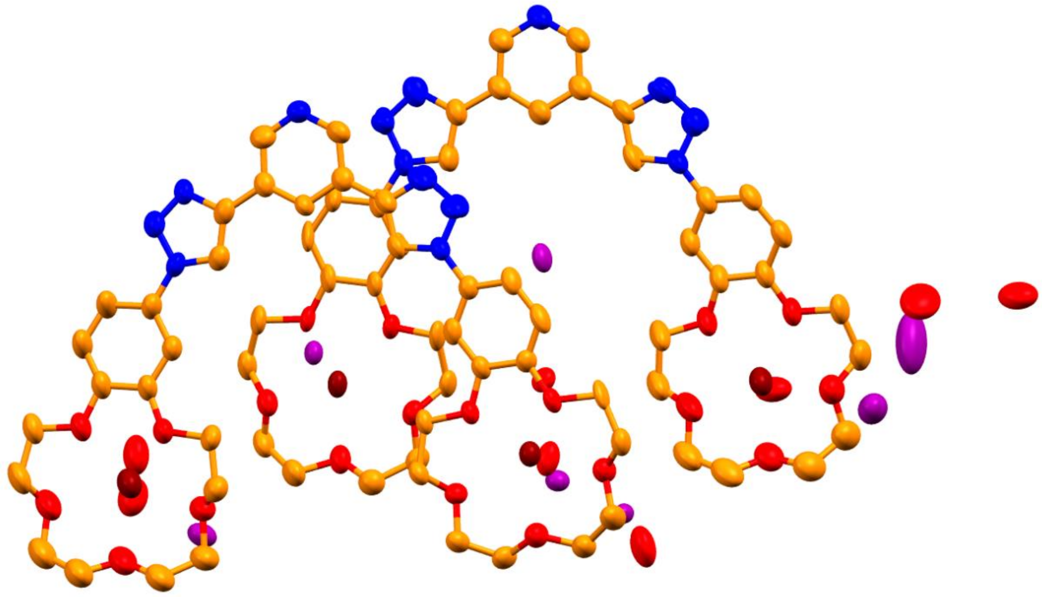

Figure S31. Thermal ellipsoid representation of the contents of the asymmetric unit for the solid state structure of the complex **1•HB**•2NaI. Ellipsoids are shown at the 50% probability level. For clarity, hydrogen atoms have been omitted.

Chloride complex of the protonated halogen-bonding receptor, 1•XB•HCl

X-ray quality crystals of the complex **1•XB**•HCl were obtained by slow evaporation of a dichloroethane/acetone solution of the receptor **1•XB**. The receptor crystallised as its hydrogen chloride salt, **1•XB**•HCl, presumably owing to the presence of a low concentration of hydrogen chloride in the dichloroethane solvent. The crystals were small and weakly diffracting and the data were therefore collected using synchrotron radiation. However, the crystal suffered severe radiation damage during data collection. As a consequence, it was only possible to obtain an incomplete set of low angle data from an initial phi scan. Despite several attempts, we were unable to obtain a more complete set of data. Nevertheless, it was possible to obtain a structure solution, which is presented here solely as a provisional guide to overall conformation and connectivity: while the overall structure is not in doubt, detailed inferences about bond lengths and angles cannot be drawn owing to the low quality of the data. Restraints to the geometries and ellipsoid parameters of all non-hydrogen atoms were applied in order to obtain a sensible refinement. Hydrogen atoms were inserted at calculated positions, refined against the data using soft restraints on bond lengths and angles and then included in the refinement using a riding model. Absent high angle data were removed after consulting the Wilson Plot.

The complex crystallised in the triclinic space group $P\bar{1}$. The asymmetric unit contains the protonated ditopic halogen-receptor 1•XB, a chloride counteranion and a partially occupied dichloroethane solvent molecule. The chloride counteranion can be seen to interact with the receptor's *N*-protonated 5-bis-iodotriazole pyridinium cavity via bidentate C—I·····Cl^- halogen bonding interactions (Figure S32). The dichloroethane solvent molecule appears to be involved in four C—H·····O^- hydrogen bonding interactions with the oxygen atoms from a proximal crown ether group. The other crown ether group forms close contacts with the *N*-protonated pyridinium group from an adjacent molecule, which strongly implies the existence

of intermolecular N—H·····O⁻ hydrogen bonding interactions. These interactions link the molecules in an infinite linear chain which is aligned with the crystallographic b axis (Figure S33). Although the electron density associated with the *N*-pyridinium proton could not be observed in the difference map its presence was inferred from the observation of this infinite linear chain, and from the presence of the chloride counteranion.

Selected crystallographic data for the **1•XB**•HCl structure are included in Table S1 below but, since the data are not of publication quality, this provisional structure has not been deposited with the Cambridge Crystallographic Data Centre, and full data in cif format are not included.

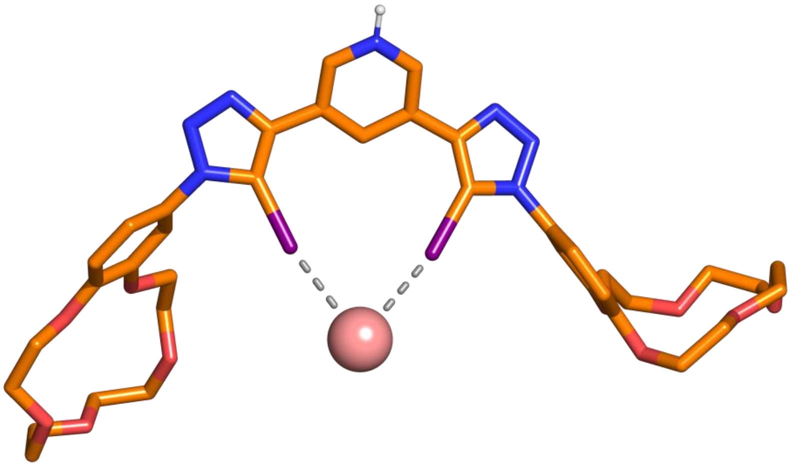

Figure S32. Content of the asymmetric unit for the solid state structure of the complex **1•XB** •HCl: the chloride counteranion can be seen to interact with the receptor's *N*-protonated bis-iodotriazole pyridinium cavity via bidentate C—I·····Cl⁻ halogen bonding interactions. For clarity, non-polar hydrogen atoms and a partially occupied dichloroethane solvent molecule have been omitted.

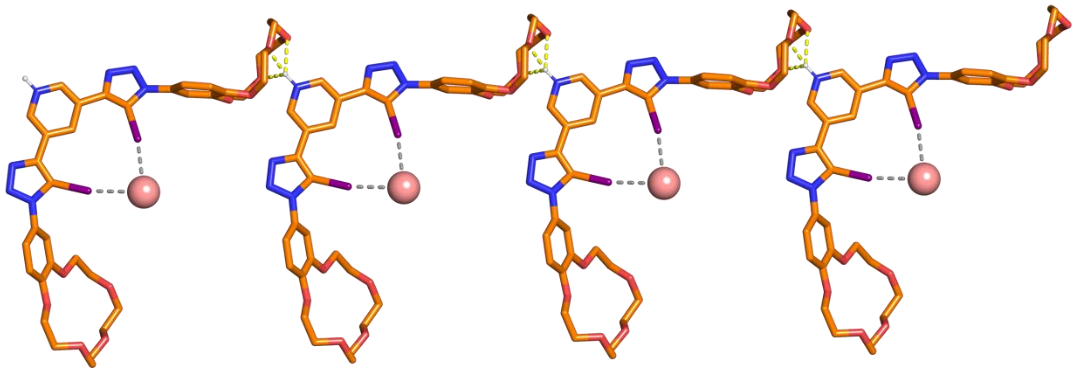

Figure S33. View of the solid state structure of the complex **1•XB** •HCl, showing the infinite linear chain which forms as a result of hydrogen-bonding interactions between *N*-protonated pyridinium and crown ether groups in adjacent molecules. For clarity, non-polar hydrogen atoms and a partially occupied dichloroethane solvent molecule have been omitted.

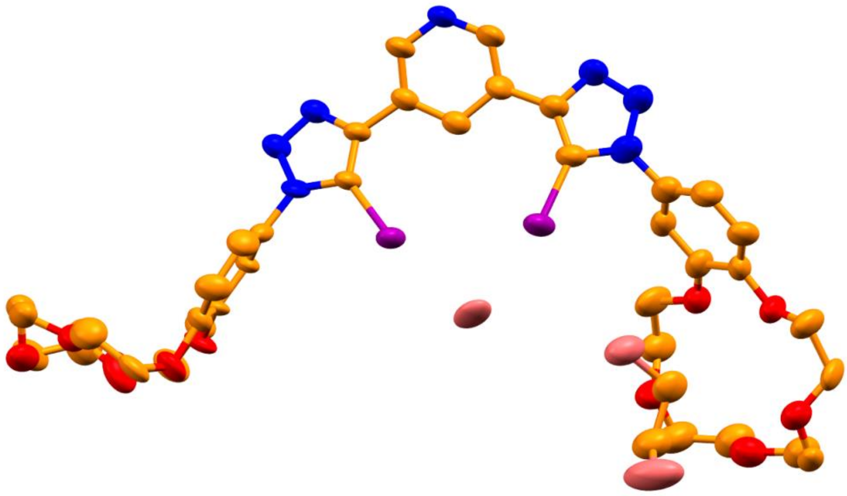

Figure S34. Thermal ellipsoid representation of the contents of the asymmetric unit for the solid state structure of the complex **1•XB**•HCl. Ellipsoids are shown at the 50% probability level. For clarity, hydrogen atoms have been omitted.

Table S1. Selected crystallographic data for the structures **1•HB**•2NaI and **1•XB**•HCl.

Compound	**1•HB**•2NaI	**1•XB**•HCl
CCDC number	1979383	—
Formula	$2(C_{37}H_{43}N_7Na_2O_{10})\cdot5.39(H_2O)\cdot4I$	$C_{37}H_{42}I_2N_7O_{10}\cdot0.72(C_2H_4Cl_2)\cdot Cl$
Formula weight	2188.29	1105.31
a (Å)	8.2042(2)	9.2629(6)
b (Å)	23.9836(7)	14.0190(9)
c (Å)	24.5580(7)	18.8620(17)
α (°)	108.317(3)	71.859(7)
β (°)	96.490(2)	77.933(7)
γ (°)	90.532(2)	78.934(5)
Unit vell volume (Å^3)	4552.8(2)	2254.9(3)
Crystal system	Triclinic	Triclinic
Space group	$P\bar{1}$	$P\bar{1}$
Z	2	2
Temperature (K)	100	100
Radiation type	Synchrotron	Synchrotron
λ (Å)	0.6889	0.6889
Reflections (all)	34930	8786
Reflections (unique)	17680	5502
R_{int}	0.086	0.139
$R[I > 2\sigma(I)]$	0.076	0.141
$wR(F^2)$ (all data)	0.221	0.369
S	0.99	1.04

References for Section 4

[S1] H. Nowell, S. A. Barnett, K. E. Christensen, S. J. Teat, D. R. Allan, *J. Synchrotron Radiat.* **2012**, *19*, 435–441.

[S2] *CrysAlisPro*, Agilent Technologies, Yarnton, Oxfordshire, UK.

[S3] L. Palatinus, G. Chapuis, *J. Appl. Crystallogr.* **2007**, *40*, 786–790.

[S4] P. W. Betteridge, J. R. Carruthers, R. I. Cooper, K. Prout, D. J. Watkin, *J. Appl. Crystallogr.* **2003**, *36*, 1487.

[S5] R. I. Cooper, A. L Thompson, D. J. Watkin, *J. Appl. Crystallogr*. **2010**, *43*, 1100–1107.

5. DFT studies

Density functional studies were performed to gain insights into the electronic structures related to Na^+ enhanced halide anion binding to the sigma hole type receptors. Overall, the calculated binding energies (Table S2) are in the same trend as the association constant from the experiment (Table 1). With the sodium cation complexation to the crown ethers, the calculated relative free energies for the binding of an iodide to [**1•XB** +2Na]$^{2+}$ and [**1•ChB** +2Na]$^{2+}$ (X = I and Ch = Te) are greatly increased in comparison to that to [**1•XB**]^0 and [**1•ChB**]^0.

Generally iodide anion is expected to attract to dication ([**1•XB** +2Na]$^{2+}$ and [**1•ChB** -2Na]$^{2+}$) more strongly than to neutral species ([**1•XB**]^0 and [**1•ChB**]^0). The X···I and the Te···I distances in [**1•XB** +2Na+I]$^{1+}$ and [**1•ChB** +2Na+I]$^{1+}$ are shorter than the corresponding distances in [**1•XB** +I]$^{1-}$ and [**1•ChB** +I]$^{1-}$ by about 0.1 Å (Table S3). From the molecular electrostatic potential (ESP) plots, the maximum ESPs for [**1•XB**]^0 and [**1•ChB**]^0 are 0.060 and 0.061 au, respectively (Figure S35). Upon sodium cation complexation, the maximum ESPs for [**1•XB** +2Na]$^{2+}$ and [**1•ChB** -2Na]$^{2+}$ become significantly more positive (0.152 and 0.188 au, respectively). That is the maximum ESPs in [**1•XB** +2Na]$^{2+}$ and [**1•ChB** +2Na]$^{2+}$ are higher than those in [**1•XB**]^0 and [**1•ChB**]^0. Correspondingly, the interaction with the incoming iodide anion is stronger for the sodium cation complexes.

The presence of Na^+ cation at the crown ether of [**1•XB**]^0 and [**1•ChB**]^0 induces the charge polarization as shown on the electronic structures of [**1•XB** +2Na]$^{2+}$ and [**1•ChB** -2Na]$^{2+}$ (Figure S35); the average NPA charges at the oxygen atoms of the crown ethers become more negative (Table S4). When a positive point charge of +1.0 is added in place of the calculated position of Na^+, the charge polarization in the electronic structures is even more pronounced (Figure S35). The NPA charges at the oxygen atoms also become more negative while the charges at the X and the Te atoms are evidently more positive (Table S4). That is the binding of the positive charge Na^+ cation at the crown ether polarizes the electronic structures of the complexes, which enhances the sigma hole bonding interaction.

Table S2. Relative free energies (in kcal/mol) for the binding of an iodide to the sigma hole type receptors.

Reaction	ΔG^a
[**1•XB**]^0 + I^- → [**1•XB** -I]$^{1-}$	–3.92
[**1•XB** -2Na]$^{2+}$ + I^- → [**1•XB** -2Na-I]$^{1+}$	–22.23
[**1•ChB**]^0 + I^- → [**1•ChB** -I]$^{1-}$	–4.65
[**1•ChB** -2Na]$^{2+}$ + I^- → [**1•ChB**-2Na-I]$^{1+}$	–19.94

[a]Optimization and single-point calculations were performed in $CHCl_3$ solvent.

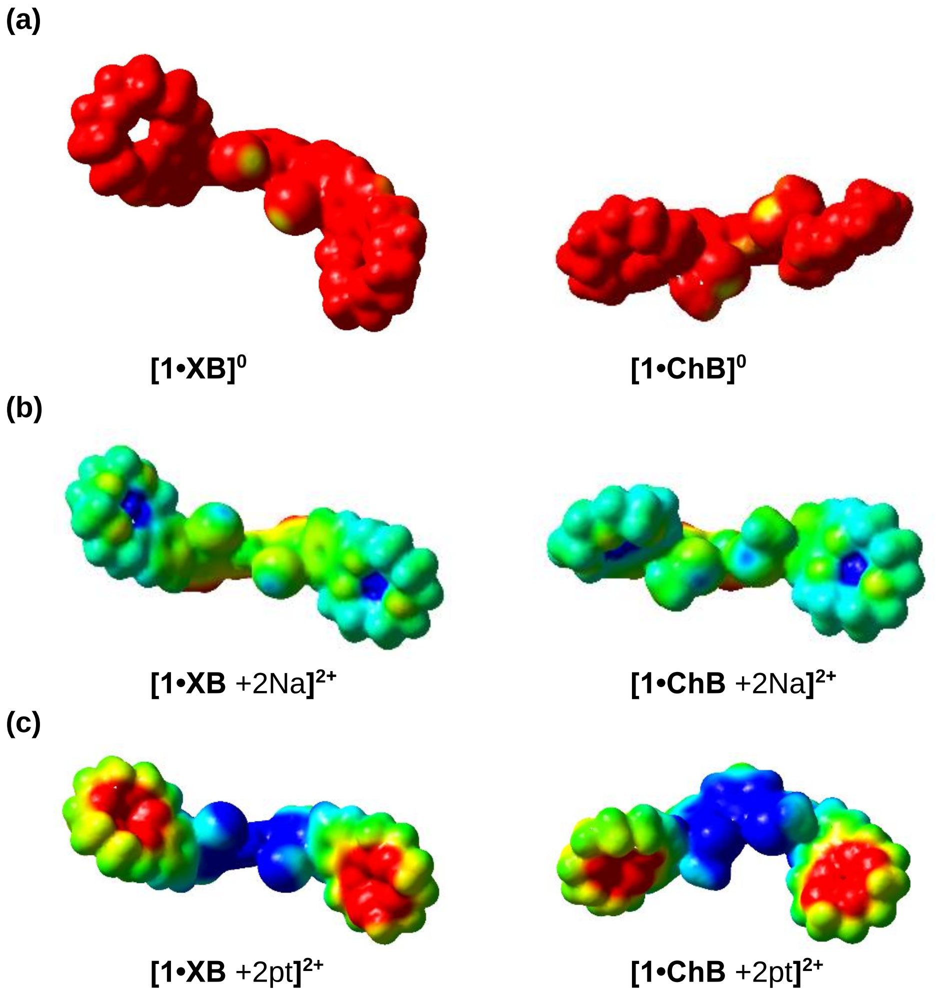

Figure S35. Electrostatic potential plots (ESPs) were mapped over electron density surfaces with isodensity of 0.004 au with the color scale from 0.05 to 0.26 au. (a) ESPs of [**1•XB**]^0 and [**1•ChB**]^0. The average maximum ESPs are 0.060 au at X and 0.061 au at Te. (b) ESPs of the complexes with two Na^+: [**1•XB** +2Na]$^{2+}$ and [**1•ChB** +2Na]$^{2+}$. The average maximum ESPs are 0.152 au at X and 0.188 au at Te. (c) ESPs of the complexes with a +1.0 point charge in place of the Na^+: **[1•XB** +2pt**]$^{2+}$** and **[1•ChB** +2pt**]$^{2+}$**. The average maximum ESPs at X are 0.262 au at X and 0.302 au at Te.

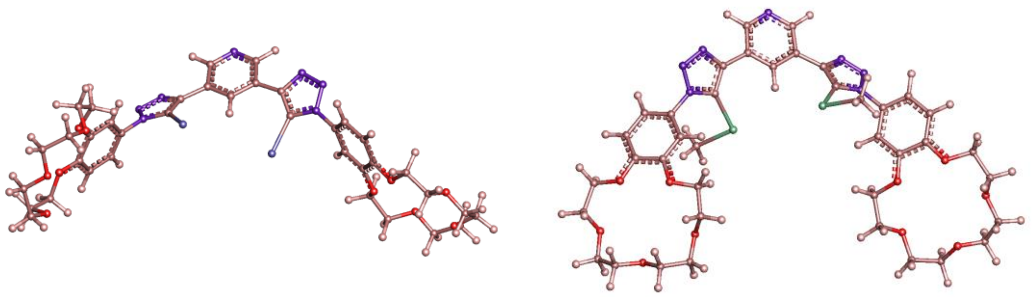

[1•XB]^{0} **[1•ChB]**^{0}

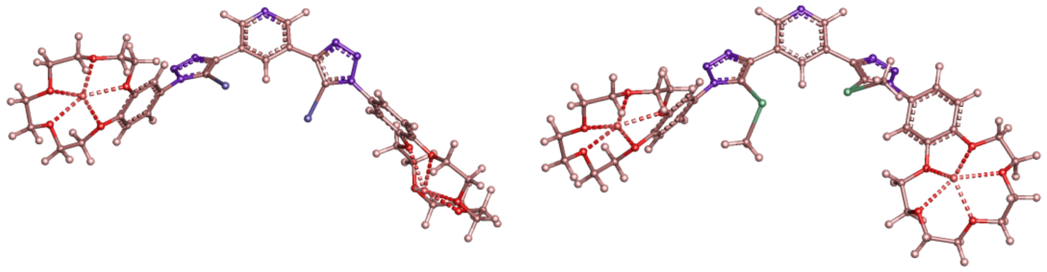

[1•XB +2Na**]**$^{2+}$ **[1•ChB** +2Na**]**$^{2+}$

Figure S36. Optimized geometries of [**1•XB**]^{0}, [**1•ChB**]^{0}, [**1•XB** +2Na]$^{2+}$, and [**1•ChB** +2Na]$^{2+}$ (X = I and Ch = Te).

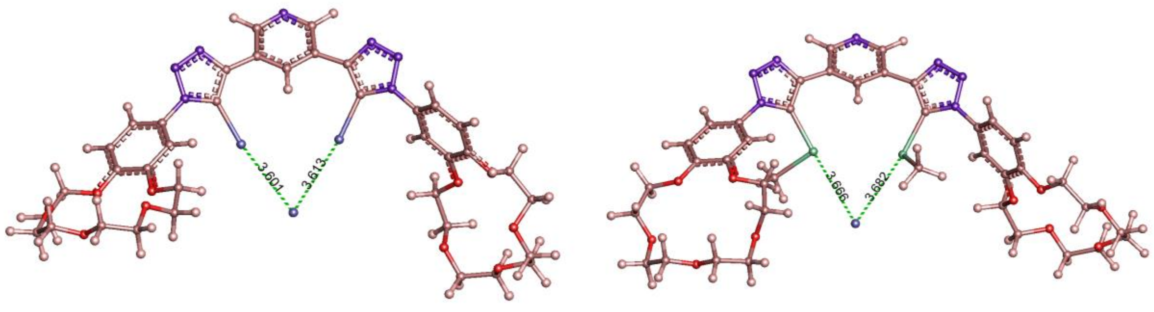

[**1•XB** +I]$^{1-}$ [**1•ChB** +I]$^{1-}$

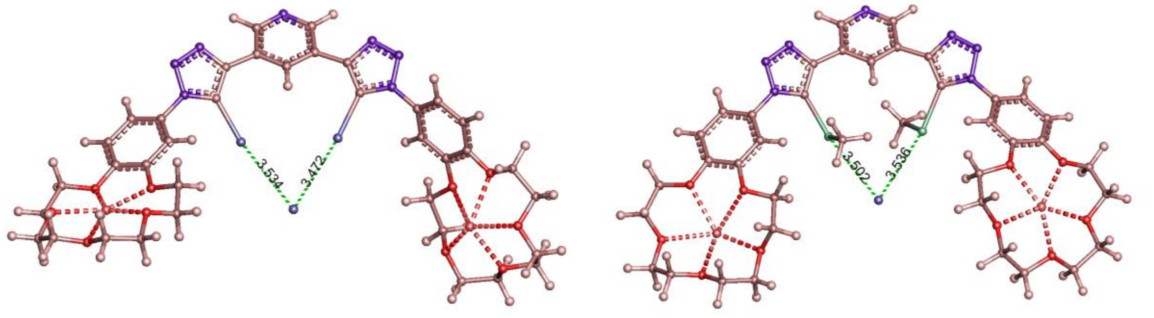

[**1•XB** +2Na+I]$^{1+}$ [**1•ChB** +2Na+I]$^{1+}$

Figure S37. Optimized geometries of [**1•XB** +I]$^{1-}$, [**1•ChB** +I]$^{1-}$, [**1•XB** +2Na+I]$^{1+}$, and [**1•ChB** +2Na+I]$^{1+}$ (X = I and Ch = Te). The X···I and Ch···I distances are shown in Å.

Table S3. Optimized geometry parameters of **1•XB** and **1•ChB** -based complexes.

Complex	average distance (Å)					average angle (°)	
	C–I	C–Te	I···I	Te···I	X···Na	C–I···I	C–Te···I
[**1•XB** +I]$^{1-}$	2.081	-	3.608	-	-	172.7	-
[**1•XB** +2Na+I]$^{1+}$	2.087	-	3.503	-	8.010	171.8	-
[**1•ChB** +I]$^{1-}$	-	2.125	-	3.674	-	-	174.8
[**1•ChB** +2Na+I]$^{1+}$	-	2.132	-	3.520	7.505	-	168.7

Table S4. Natural population analysis (NPA) charge on **1•XB** and **1•ChB** -based complexes.

Complex	Total	average charge (q)				
	charge	I	Te	Na	point charge	O (crown ether)
[**1•XB**]^{0}	0.000	0.321	-	-	-	-0.598
[**1•XB** +2Na]$^{2+}$	2.000	0.320	-	0.911	-	-0.640
[**1•XB** +2pt]$^{2+}$	2.000	0.574	-	-	1.000	-0.689
[**1•ChB**]^{0}	0.000	-	0.686	-	-	-0.597
[**1•ChB** +2Na]$^{2+}$	2.000	-	0.691	0.911	-	-0.640
[**1•ChB** +2pt]$^{2+}$	2.000	-	1.123	-	1.000	-0.693

Computational details

All the calculations were performed with Gaussian 09 program.[1] The geometry optimizations were calculated using M06-2X functional[2] and basis set 1 (BS1). The BS1 includes LANL2DZdp with effective core potentials (ECP)[3] for iodine and tellurium, and 6-31G(d)[4] for all other atoms. The single-point energy calculations were carried out subsequently for IEFPCM[5] solvent correction with $CHCl_3$ parameters using M06-2X and the basis set 2 (BS2). The BS2 includes LANL2DZdp with effective core potentials (ECP) for iodine and tellurium, and 6-31++G(d,p) for all other atoms. The molecular electrostatic potentials (MEPs) were plotted over electron density surface with an isovalue of 0.004 au. The color scale is ranged from 0.26 (blue) to 0.05 (red) au. The surface maxima on the MEP surfaces, between the most negative and most positive values, were calculated using *multiwfn* program.[6] Natural population analysis (NPA) was performed using NBO 6.0.[7]

References for Section 5

[1] M. J. Frisch, G. W. Trucks, H. B. Schlegel, G. E. Scuseria, M. A. Robb, J. R. Cheeseman, G. Scalmani, V. Barone, B. Mennucci, G. A. Petersson, H. Nakatsuji, M. Caricato, X. Li, H. P. Hratchian, A. F. Izmaylov, J. Bloino, G. Zheng, J. L. Sonnenberg, M. Hada, M. Ehara, K. Toyota, R. Fukuda, J. Hasegawa, M. Ishida, T. Nakajima, Y. Honda, O. Kitao, H. Nakai, T. Vreven, J. A. Montgomery Jr., J. E. Peralta, F. Ogliaro, M. J. Bearpark, J. Heyd, E. N. Brothers, K. N. Kudin, V. N. Staroverov, R. Kobayashi, J. Normand, K. Raghavachari, A. P. Rendell, J. C. Burant, S. S. Iyengar, J. Tomasi, M. Cossi, N. Rega, N. J. Millam, M. Klene, J. E. Knox, J. B. Cross, V. Bakken, C. Adamo, J. Jaramillo, R. Gomperts, R. E. Stratmann, O. Yazyev,

A. J. Austin, R. Cammi, C. Pomelli, J. W. Ochterski, R. L. Martin, K. Morokuma, V. G. Zakrzewski, G. A. Voth, P. Salvador, J. J. Dannenberg, S. Dapprich, A. D. Daniels, Ö. Farkas, J. B. Foresman, J. V. Ortiz, J. Cioslowski, D. J. Fox, Gaussian, Inc., Wallingford, CT, USA, **2009**.

[2] Y. Zhao, D. G. Truhlar, *Theoretical Chemistry Accounts* **2008**, *120*, 215-241.

[3] aC. E. Check, T. O. Faust, J. M. Bailey, B. J. Wright, T. M. Gilbert, L. S. Sunderlin, *The Journal of Physical Chemistry A* **2001**, *105*, 8111-8116; bP. J. Hay, W. R. Wadt, *The Journal of Chemical Physics* **1985**, *82*, 299-310; cW. R. Wadt, P. J. Hay, *The Journal of Chemical Physics* **1985**, *82*, 284-298.

[4] aP. C. Hariharan, J. A. Pople, *Theoretica Chimica Acta* **1973**, *28*, 213-222; bG. A. Petersson, M. A. Al-Laham, *The Journal of Chemical Physics* **1991**, *94*, 6081-6090; cG. A. Petersson, A. Bennett, T. G. Tensfeldt, M. A. Al-Laham, W. A. Shirley, J. Mantzaris, *The Journal of Chemical Physics* **1988**, *89*, 2193-2218; dW. J. Hehre, R. Ditchfield, J. A. Pople, *The Journal of Chemical Physics* **1972**, *56*, 2257-2261.

[5] aB. Mennucci, J. Tomasi, *The Journal of Chemical Physics* **1997**, *106*, 5151-5158; bB. Mennucci, R. Cammi, J. Tomasi, *The Journal of Chemical Physics* **1998**, *109*, 2798-2807.

[6] T. Lu, F. Chen, *Journal of Computational Chemistry* **2012**, *33*, 580-592.

[7] aA. E. Reed, F. Weinhold, *The Journal of Chemical Physics* **1985**, *83*, 1736-1740; bA. E. Reed, L. A. Curtiss, F. Weinhold, *Chemical Reviews* **1988**, *88*, 899-926.

XYZ coordinates

[1•XB]^0
97

C	-6.70292200	-1.34861700	-1.29587900
O	-7.61294600	-2.33264300	-1.15032300
C	-8.15443400	-2.92355300	-2.31982600
C	-9.30087900	-3.80280800	-1.87646100
O	-10.28635700	-2.97727900	-1.31371900
C	-11.33146900	-3.68369200	-0.68730100
C	-12.01356900	-2.74635500	0.28463600
O	-11.11728300	-2.50245800	1.33940900
C	-11.55216300	-1.51193200	2.23697100
C	-10.41332100	-1.22963100	3.19178700
O	-9.38860700	-0.59069100	2.46756500
C	-8.12744400	-0.66648800	3.07829000
C	-7.10510600	-0.12919200	2.10288000
O	-7.09974400	-1.00011000	0.98400200
C	-6.41872200	-0.61448000	-0.11510500
C	-5.50511600	0.42631900	-0.15654500
C	-4.85930700	0.72034900	-1.36294000
C	-5.12740300	0.01855000	-2.52139600
C	-6.06269600	-1.02031200	-2.48377500
N	-3.91830100	1.79619200	-1.40256900
N	-4.14083200	2.84996300	-2.20038600
N	-3.15438500	3.67099300	-2.06071100
C	-2.25639200	3.17251500	-1.16381100
C	-2.74477100	1.95018100	-0.73225400
C	-1.05411700	3.94099000	-0.81607200
C	-1.06924900	5.33151100	-0.98468800

N	-0.03340100	6.12097800	-0.70580200
C	1.07806900	5.54991100	-0.24490000
C	1.21480700	4.17144100	-0.04205100
C	0.11883900	3.36076800	-0.33470700
C	2.48541800	3.65284500	0.47951500
C	3.12498500	2.43344900	0.33153000
N	4.28426400	2.57084700	1.02980000
N	4.35747200	3.79243400	1.57629700
N	3.28826700	4.43981700	1.25070700
C	5.34268600	1.63254100	1.23460900
C	6.03867800	1.14395300	0.12360500
C	7.05737100	0.22462700	0.31407000
C	7.39898000	-0.18525900	1.62820000
C	6.70824100	0.33616400	2.71441100
C	5.66498500	1.24741900	2.52063500
O	8.40265400	-1.08298100	1.70141700
C	8.67147700	-1.68302000	2.95739800
C	9.67278200	-2.78613900	2.70949300
O	9.03007800	-3.79858500	1.97299000
O	9.43780100	-4.09818900	-0.86991800
C	9.87440900	-3.56346800	-2.09505200
C	8.67528900	-3.39167700	-3.00904600
O	7.77151000	-2.41190100	-2.54463500
C	8.14425800	-1.09519400	-2.87136200
C	7.33638100	-0.15587900	-2.00821200
O	7.78538400	-0.33403900	-0.67524600
I	-2.00373500	0.53675000	0.57680300
I	2.59498400	0.69767600	-0.65134200
H	-8.51745900	-2.14256200	-2.99976100
H	-7.39622300	-3.52481500	-2.83823900
H	-9.68611100	-4.36136700	-2.74409000
H	-8.93664900	-4.53002700	-1.13562500
H	-12.05310400	-4.05877900	-1.42878600
H	-10.93397700	-4.54434100	-0.12932300
H	-12.94615200	-3.20168200	0.65457000
H	-12.27191000	-1.80856400	-0.23063300
H	-12.43906000	-1.84689900	2.79826100
H	-11.81714700	-0.58923800	1.69860300
H	-10.75852700	-0.59951300	4.02554200
H	-10.05439000	-2.18274100	3.60711100
H	-8.08958100	-0.07352700	4.00551800
H	-7.88101800	-1.70897600	3.32833700
H	-7.38291600	0.88411000	1.78584400
H	-6.11397200	-0.09683500	2.57526000
H	-5.29225700	1.02035300	0.72472700
H	-4.61944200	0.27716200	-3.44344700
H	-6.28149300	-1.57029900	-3.39102000
H	-1.96763200	5.81401600	-1.36043700
H	1.91368000	6.20815600	-0.02162100
H	0.17954100	2.28985300	-0.18656100
H	5.77924300	1.49306600	-0.86890800
H	6.96401900	0.03204300	3.72204800
H	5.11337400	1.64827200	3.36337900
H	7.74971800	-2.10255000	3.37939300
H	9.08271600	-0.94446700	3.65756500

H	10.03896700	-3.17582000	3.67144500
H	10.52970700	-2.36784700	2.16351500
H	10.60385200	-4.23619800	-2.57587200
H	10.37731100	-2.59762000	-1.92843100
H	8.12054900	-4.33379200	-3.04334000
H	9.01917300	-3.15593900	-4.02731700
H	7.94514500	-0.88474600	-3.93317600
H	9.21179800	-0.91661500	-2.68313000
H	6.26917400	-0.40368000	-2.08108500
H	7.48611200	0.88272000	-2.33114900
C	10.48493500	-4.21065400	0.06150200
H	11.25346200	-4.91500600	-0.29775400
H	10.97382200	-3.23366000	0.20212800
C	9.91584700	-4.71889700	1.37322300
H	10.73833500	-4.97615700	2.05746200
H	9.33546900	-5.62532200	1.17767900

[1•ChB]^{0}
105

C	-6.91137900	0.00489500	0.63966300
O	-7.76161400	0.98986600	0.99642100
C	-9.12854300	0.65833600	1.17876400
C	-9.89279400	1.95965700	1.26648900
O	-9.77422100	2.61657200	0.03219200
C	-10.32660500	3.91170500	0.02256200
C	-9.73232900	4.66190200	-1.14880800
O	-8.37313700	4.88094500	-0.86609700
C	-7.64389400	5.40366000	-1.94807200
C	-6.17831600	5.35585900	-1.57661400
O	-5.78492800	4.00456800	-1.53748900
C	-4.60793300	3.77722100	-0.80805000
C	-4.41322400	2.28231000	-0.69931500
O	-5.50192000	1.77967600	0.05647300
C	-5.66221600	0.44182800	0.12648300
C	-4.71984000	-0.49211800	-0.27592400
C	-5.00732700	-1.85511900	-0.14227800
C	-6.22182200	-2.29544800	0.34678500
C	-7.18140300	-1.35416500	0.73154000
N	-4.01108400	-2.81177000	-0.50447100
N	-4.30571000	-3.76956600	-1.39609000
N	-3.26025200	-4.51454700	-1.53486900
C	-2.26552600	-4.05772400	-0.72226900
C	-2.73978300	-2.95057700	-0.02922900
C	-0.96762800	-4.73772600	-0.67416500
C	-0.88657700	-6.10632000	-0.96016800
N	0.25019300	-6.80081700	-0.93543800
C	1.37197900	-6.15167100	-0.62694500
C	1.42132300	-4.78410200	-0.33076500
C	0.21891400	-4.07924900	-0.35477500
C	2.70539100	-4.15078000	-0.01701400
C	3.15072000	-2.85178800	-0.22964700
N	4.42380300	-2.87962700	0.25990200
N	4.74600900	-4.10146100	0.71131600
N	3.71672100	-4.86479800	0.55374400

C	5.39702200	-1.83593200	0.29189800
C	5.07264600	-0.62598800	0.91476300
C	5.98401600	0.41767800	0.88516000
C	7.24504800	0.23398600	0.26177300
C	7.55564600	-0.99114300	-0.31484700
C	6.62299700	-2.03278100	-0.31310900
O	8.05831100	1.30983100	0.28622400
C	9.25007100	1.25807200	-0.48034600
C	9.83321600	2.65047700	-0.47878400
O	8.97695400	3.47538100	-1.23160400
C	9.19923100	4.85586300	-1.04206900
C	8.51953000	5.39127400	0.20465200
O	7.12737800	5.28821500	0.03703800
C	6.42291600	5.69603900	1.18310800
C	4.94027300	5.48311300	0.94031500
O	4.60357400	4.11838200	0.81222000
C	4.42269300	3.46247000	2.04448900
C	4.44464100	1.97405200	1.79169800
O	5.77167100	1.63844900	1.42114600
Te	-1.87856900	-1.78730800	1.49441800
Te	2.23796300	-1.21636700	-1.18359300
H	-9.48600100	0.06834900	0.32551600
H	-9.26877700	0.07737500	2.09966600
H	-10.94485400	1.74411100	1.51089200
H	-9.47373000	2.57727400	2.07512400
H	-11.42314500	3.87162400	-0.06433200
H	-10.07481100	4.44503800	0.95127900
H	-10.26582500	5.61451100	-1.29598500
H	-9.84595100	4.06118900	-2.06406100
H	-7.94372700	6.44135200	-2.16470000
H	-7.81106000	4.80319300	-2.85520100
H	-5.57912600	5.92011100	-2.30755500
H	-6.04651100	5.82401400	-0.58996200
H	-3.73083500	4.22263900	-1.30341100
H	-4.68833500	4.21046200	0.20012000
H	-4.41201600	1.82975800	-1.69926300
H	-3.45897800	2.05863700	-0.20354500
H	-3.76780700	-0.18406400	-0.69087800
H	-6.41793600	-3.35803300	0.43441700
H	-8.13661500	-1.69539600	1.11225700
H	-1.79135500	-6.65258300	-1.21419600
H	2.29045100	-6.73292600	-0.61664300
H	0.20688300	-3.01937200	-0.12260800
H	4.11152400	-0.51545900	1.40193200
H	8.51813000	-1.14353000	-0.78785000
H	6.84701900	-2.98136200	-0.78759500
H	9.02353000	0.95369200	-1.50988200
H	9.95903000	0.54339100	-0.04316000
H	10.84176500	2.62911900	-0.91915200
H	9.92225400	2.99706800	0.55998800
H	10.27593700	5.07847900	-0.99940500
H	8.81825000	6.44155600	0.35827200
H	6.60731400	6.76200400	1.39661000
H	6.75895400	5.12154100	2.06099800
H	4.66567400	5.96121700	-0.00454600

H	4.36201900	5.95603800	1.74801400
H	3.46109000	3.74575300	2.49923600
H	5.22182900	3.71114500	2.75655800
H	3.75116300	1.71972100	0.97871600
H	4.14729300	1.42963000	2.69745200
H	8.77869200	5.35978200	-1.91711700
H	8.83754200	4.82579700	1.09485500
C	3.93511700	-0.68274700	-2.37893000
C	-3.59268600	-1.82419400	2.78125600
H	3.55133600	-0.06669300	-3.19492200
H	4.38033500	-1.59054600	-2.78710100
H	4.66079300	-0.12002200	-1.79201400
H	-3.97950900	-2.84238300	2.83018200
H	-4.35685700	-1.13363100	2.42463300
H	-3.23920200	-1.52035100	3.76898200

[1•XB +2Na**]$^{2+}$**
99

C	7.13029800	1.44598700	-1.26300500
O	8.19527800	2.28266000	-1.08784000
C	8.72958500	2.95004300	-2.23427900
C	9.97958000	3.67772000	-1.78829600
O	10.87633100	2.72821000	-1.23712700
C	12.06390500	3.29936000	-0.70224300
C	12.76859800	2.22978000	0.10526900
O	11.88935500	1.84277700	1.15017400
C	12.33181900	0.73891900	1.92494600
C	11.20181900	0.36634600	2.86157200
O	10.08416400	-0.00943500	2.06573800
C	8.87259000	-0.12642400	2.79193000
C	7.76304200	-0.43471600	1.80965900
O	7.74728900	0.62606800	0.85263300
C	6.88830700	0.54138100	-0.20473400
C	5.85073600	-0.37010300	-0.29596900
C	5.03864300	-0.35786900	-1.43395000
C	5.25860600	0.52586900	-2.47430400
C	6.31878300	1.43079400	-2.38751500
N	3.99618000	-1.32458500	-1.55223100
N	4.00989300	-2.18423100	-2.58419300
N	2.99537600	-2.97133100	-2.46011900
C	2.28809200	-2.64330100	-1.34162300
C	2.93032500	-1.57433500	-0.74404900
C	1.10720700	-3.41778400	-0.94001500
C	1.10968200	-4.80326300	-1.14072500
N	0.10091800	-5.60179400	-0.79716200
C	-0.97088600	-5.03976200	-0.24172900
C	-1.09596300	-3.66458100	-0.01385100
C	-0.02743300	-2.84271000	-0.36920600
C	-2.33080900	-3.15643600	0.59617000
C	-3.08023600	-2.02315800	0.34013900
N	-4.13734700	-2.11655400	1.19092700
N	-4.04569800	-3.23566100	1.92728000
N	-2.97157100	-3.85780000	1.57382300
C	-5.25465000	-1.24714300	1.37049700

C	-6.11562800	-1.01584400	0.29128000
C	-7.21449500	-0.20171300	0.49054700
C	-7.45594500	0.37281600	1.75774100
C	-6.59340500	0.12792400	2.81327600
C	-5.47908000	-0.69349400	2.61702700
O	-8.57188100	1.14844400	1.80514700
C	-8.93271000	1.78545000	3.02614100
C	-10.30560400	2.37452100	2.79653000
O	-10.22426200	3.25523400	1.68773900
O	-11.24392800	2.76551400	-0.85295700
C	-11.69596600	1.91776300	-1.89641200
C	-10.61773500	1.91476500	-2.96276100
O	-9.37910900	1.44096600	-2.44128700
C	-9.22366900	0.03365200	-2.53228700
C	-7.96708000	-0.35015800	-1.78585500
O	-8.14452600	0.11435000	-0.45200100
Na	-9.08485000	2.31708000	-0.21764100
Na	9.82263300	1.42267000	0.30704400
I	2.49035600	-0.50914700	0.96613200
I	-2.79503800	-0.47657600	-0.99443100
H	8.95934600	2.20796400	-3.00755900
H	8.00908100	3.67499600	-2.62550600
H	10.42882100	4.18249800	-2.65252600
H	9.72706800	4.43401900	-1.03204800
H	12.71441000	3.65603700	-1.50981000
H	11.80633900	4.14727600	-0.05252400
H	13.70538700	2.62673600	0.51495300
H	12.99942700	1.36276500	-0.52942500
H	13.22569900	1.00457000	2.50235000
H	12.57390500	-0.10767800	1.26700800
H	11.50500900	-0.46336300	3.51118200
H	10.93682600	1.22980600	3.48676800
H	8.93642800	-0.93488800	3.53072400
H	8.66028200	0.81523900	3.31775600
H	7.94760300	-1.38480100	1.29471400
H	6.80609700	-0.48387300	2.34038900
H	5.67690100	-1.10369900	0.48217600
H	4.62085800	0.50333000	-3.35009000
H	6.49985700	2.11623400	-3.20653500
H	1.97730600	-5.27535500	-1.59529400
H	-1.78541200	-5.70354100	0.03813400
H	-0.07871000	-1.77148100	-0.20103500
H	-5.92258600	-1.48401400	-0.66715300
H	-6.76852100	0.57032900	3.78676800
H	-4.79365400	-0.89899100	3.43087500
H	-8.19900000	2.56373500	3.26649900
H	-8.97574500	1.05566400	3.84154200
H	-10.62821700	2.91851800	3.69276500
H	-11.02134900	1.56557800	2.59840400
H	-12.63682900	2.29515600	-2.31716200
H	-11.87362400	0.90627000	-1.50264100
H	-10.43839500	2.93831700	-3.30131500
H	-10.92992400	1.31138200	-3.82271300
H	-9.13779700	-0.27371300	-3.58174100
H	-10.08359600	-0.48068500	-2.08333700

H	-7.07906500	0.12139800	-2.22440000
H	-7.84944600	-1.43881800	-1.80191100
C	-12.13550300	2.85839500	0.24650200
H	-13.09701800	3.28057500	-0.07336100
H	-12.31781100	1.85692800	0.66306700
C	-11.48458500	3.77016100	1.26920700
H	-12.14740400	3.91402900	2.13024100
H	-11.28539800	4.74386600	0.81442300

[1•ChB +2Na**]$^{2+}$**
107

C	6.79554400	-0.67351800	1.50858500
O	7.70937900	-1.68812800	1.55078400
C	8.54000200	-1.80147700	2.70984300
C	9.55835700	-2.88554800	2.43065200
O	10.28516100	-2.52229400	1.26948900
C	11.22574100	-3.50091800	0.84501200
C	11.71245700	-3.10138700	-0.53127700
O	10.58375000	-3.09140400	-1.39213000
C	10.83162500	-2.56726900	-2.68761600
C	9.49883000	-2.47675800	-3.39965800
O	8.66982500	-1.59162000	-2.65651400
C	7.30888700	-1.60803500	-3.05039600
C	6.54433500	-0.67755400	-2.13432800
O	6.73984700	-1.14706500	-0.79776500
C	6.26667500	-0.38209800	0.23077700
C	5.35054400	0.64530600	0.07723100
C	4.94736300	1.36600000	1.20485200
C	5.45865800	1.09321700	2.46068900
C	6.39394000	0.06714500	2.61047300
N	4.00772700	2.42763100	1.05231400
N	4.36647400	3.66805000	1.41632400
N	3.36401800	4.44653000	1.19338000
C	2.31955300	3.72082600	0.69624500
C	2.71938700	2.39537000	0.59905800
C	1.06320700	4.40767500	0.35716100
C	1.12614600	5.74765800	-0.04585400
N	0.05631600	6.49289200	-0.31998500
C	-1.14052600	5.92118900	-0.20837500
C	-1.33038600	4.58950900	0.17961200
C	-0.20059600	3.82746600	0.47613400
C	-2.69605100	4.06722600	0.29425700
C	-3.22145000	2.83389200	-0.06095900
N	-4.54303800	2.95837900	0.25817400
N	-4.81148100	4.18084600	0.74690200
N	-3.70701700	4.84601200	0.77547600
C	-5.61456700	2.03401700	0.09140300
C	-5.50579100	0.75065100	0.64112500
C	-6.55027800	-0.13581800	0.45141600
C	-7.70353000	0.25994600	-0.26062100
C	-7.79906200	1.53901100	-0.78164300
C	-6.73997000	2.43478400	-0.60660900
O	-8.64443300	-0.71629300	-0.36576600
C	-9.87881600	-0.43783300	-1.01789300

C	-10.75965000	-1.63647400	-0.75066400
O	-10.08991600	-2.78468600	-1.24597000
C	-10.75455800	-4.01078600	-0.95983700
C	-10.32036800	-4.56849800	0.38216800
O	-8.91385300	-4.74027600	0.32203600
C	-8.34091100	-5.24699200	1.51550500
C	-6.84678000	-5.35209800	1.27848600
O	-6.28601200	-4.08470700	0.94920400
C	-5.90037100	-3.32001600	2.08046200
C	-5.47236700	-1.95300200	1.59823600
O	-6.59567400	-1.41666500	0.91041200
Na	-7.78462400	-2.92083000	-0.53615300
Na	8.85239500	-1.98447600	-0.42087000
Te	1.73612400	0.66837700	-0.08372200
Te	-2.32297200	1.21227000	-1.04652000
H	9.03252700	-0.83993200	2.89446000
H	7.94138700	-2.07998700	3.58297200
H	10.22647600	-2.98106200	3.29555900
H	9.05345700	-3.84828300	2.26870000
H	12.06776400	-3.55280100	1.54572000
H	10.74084400	-4.48600500	0.79983900
H	12.46391000	-3.81613000	-0.88793100
H	12.16395800	-2.10004400	-0.49652200
H	11.51135900	-3.22209100	-3.24668200
H	11.28693300	-1.57025100	-2.60624900
H	9.63830000	-2.10221400	-4.42073400
H	9.03213700	-3.47036000	-3.44699400
H	7.19816100	-1.26509600	-4.08677900
H	6.90957300	-2.62927000	-2.97291900
H	6.92345900	0.34785200	-2.21499900
H	5.48080600	-0.69776900	-2.39430100
H	4.95597000	0.90982100	-0.89638300
H	5.13671200	1.67736000	3.31508400
H	6.80197100	-0.14108200	3.59197000
H	2.09547000	6.23070700	-0.13662300
H	-2.00524000	6.54056600	-0.43406700
H	-0.30353000	2.81112500	0.84773000
H	-4.62083600	0.47473100	1.20237300
H	-8.67878500	1.84827000	-1.33359200
H	-6.79039300	3.43645500	-1.01716300
H	-9.70762700	-0.29967900	-2.09200000
H	-10.33765900	0.46408500	-0.59957300
H	-11.72460600	-1.50879800	-1.25621900
H	-10.93565800	-1.72515400	0.32963900
H	-11.84207800	-3.87590900	-0.98567600
H	-10.81670200	-5.53035000	0.56509100
H	-8.75269400	-6.23747600	1.74941500
H	-8.56705900	-4.57230000	2.35434200
H	-6.65883500	-6.00237500	0.42025200
H	-6.34819100	-5.77754200	2.15733600
H	-5.06795800	-3.80668500	2.60321400
H	-6.74094900	-3.21101800	2.77880200
H	-4.61643600	-2.01785900	0.91502600
H	-5.20900700	-1.32460800	2.45565800
H	-10.46982400	-4.70715700	-1.75280900

H	-10.57674600	-3.88490200	1.20462800
C	-3.88165400	1.00540300	-2.50697600
C	2.24796100	-0.52037400	1.62631000
H	-3.46695500	0.40439800	-3.31937700
H	-4.14897800	1.99224200	-2.88603600
H	-4.75123100	0.50711900	-2.07803500
H	2.07536600	0.06206600	2.53180200
H	3.28578700	-0.84917000	1.56884500
H	1.58148300	-1.38503000	1.61677200

[**1•XB** +I]$^{1-}$
98

C	-7.20436700	-0.11826500	-0.83091100
O	-8.07314300	-1.13494400	-1.02242500
C	-9.35653400	-0.82629400	-1.53487300
C	-10.20323600	-2.06965300	-1.38457500
O	-10.34123900	-2.34070400	-0.01385700
C	-10.97583100	-3.56777200	0.25942800
C	-10.63841700	-3.96703900	1.67925900
O	-9.27684000	-4.31244300	1.71318300
C	-8.76523200	-4.49814300	3.00944300
C	-7.26003500	-4.60531600	2.89775000
O	-6.75754500	-3.33752700	2.54953600
C	-5.46589600	-3.37249700	1.99764900
C	-5.13377600	-1.98220500	1.50710800
O	-6.06396600	-1.68038700	0.48222000
C	-6.09439600	-0.42203000	-0.00244600
C	-5.15131700	0.55659500	0.26912200
C	-5.29491200	1.82456000	-0.30624800
C	-6.37025400	2.13424600	-1.11672100
C	-7.33411000	1.15383800	-1.37076300
N	-4.32700800	2.83481700	-0.02294900
N	-4.73577000	4.03095400	0.43205600
N	-3.68975500	4.77131400	0.59528000
C	-2.57232100	4.06805700	0.25499400
C	-2.97371200	2.80740100	-0.15703600
C	-1.24373600	4.69021800	0.32872200
C	-1.12980400	6.06810600	0.10947200
N	0.01753300	6.73997900	0.19279000
C	1.11655900	6.04967200	0.49320100
C	1.13067200	4.67000300	0.73220800
C	-0.08360000	3.98550000	0.65602700
C	2.40314300	4.03252200	1.09940500
C	2.92248800	2.78014000	0.81402200
N	4.14863500	2.79071100	1.40444100
N	4.37156400	3.96849500	2.01229200
N	3.33206300	4.71262500	1.82978900
C	5.14534200	1.77146200	1.48282000
C	5.60455900	1.16103400	0.31116100
C	6.57828600	0.17834400	0.39264500
C	7.12654500	-0.16736500	1.65248000
C	6.66816400	0.46883500	2.79888000
C	5.66420400	1.43828900	2.71960200
O	8.08273700	-1.12129900	1.62103700

C	8.58618300	-1.59513300	2.85663700
C	9.53197000	-2.72817300	2.53773100
O	8.77195100	-3.80277000	2.04127700
O	8.57105600	-4.37384600	-0.79153600
C	8.72614400	-3.97705100	-2.13202800
C	7.35193800	-3.84201700	-2.76225300
O	6.61058400	-2.77505000	-2.21444900
C	6.91513700	-1.52280500	-2.77851600
C	6.34242500	-0.45224300	-1.88074900
O	7.07921000	-0.49247600	-0.66771400
I	-1.88691400	1.24404000	-0.99787200
I	2.11270500	1.19352500	-0.26140300
H	-9.79727100	0.00296900	-0.96663100
H	-9.29695100	-0.54042200	-2.59328600
H	-11.18089500	-1.90279100	-1.86378400
H	-9.71037700	-2.90980300	-1.89561800
H	-12.06649600	-3.48213500	0.13582300
H	-10.61375400	-4.34968400	-0.42444200
H	-11.27049100	-4.81402300	1.99098500
H	-10.84230100	-3.12216300	2.35462200
H	-9.17567400	-5.41098300	3.47000000
H	-9.02253000	-3.64362100	3.65342100
H	-6.83025000	-4.95231000	3.85007600
H	-7.01347000	-5.34491600	2.12157300
H	-4.71634000	-3.68483100	2.74149800
H	-5.42586400	-4.07739900	1.15427300
H	-5.23316300	-1.25929900	2.32723100
H	-4.10586800	-1.94974200	1.12259500
H	-4.31270900	0.35581300	0.92510800
H	-6.46086900	3.12670900	-1.54258300
H	-8.18181300	1.39454100	-2.00120000
H	-2.01906000	6.64252400	-0.13785300
H	2.04632500	6.60987400	0.55654400
H	-0.13385600	2.92581700	0.88756500
H	5.20379500	1.46589400	-0.64769700
H	7.07406000	0.20817200	3.76868900
H	5.29445700	1.92789100	3.61298200
H	7.76443000	-1.95927700	3.48629600
H	9.11730100	-0.79536100	3.38944500
H	10.07693100	-3.02342100	3.44759800
H	10.26691000	-2.37571800	1.80081100
H	9.31663200	-4.72115300	-2.69226300
H	9.26830200	-3.01939500	-2.18335300
H	6.78120200	-4.75347200	-2.56225500
H	7.45492800	-3.73074500	-3.85209500
H	6.47792800	-1.43081100	-3.78421700
H	8.00057000	-1.37041100	-2.86331900
H	5.28165000	-0.65343600	-1.68300300
H	6.43822800	0.53073200	-2.35996400
C	9.79260200	-4.37856600	-0.09645500
H	10.49522100	-5.10119700	-0.54434500
H	10.26308800	-3.38364100	-0.15177800
C	9.53236500	-4.76742200	1.34734200
H	10.48993600	-4.95320200	1.85680900
H	8.94939200	-5.69280800	1.36333100

I	0.36632200	-1.27957400	-2.23266600

[**1•ChB** +I]$^{1-}$
106

C	6.53192900	-0.57216000	1.74742000
O	7.36475000	-1.63341700	1.82975400
C	8.10464600	-1.80563700	3.02464000
C	9.13227900	-2.88129500	2.75632500
O	10.01041300	-2.40554500	1.76977600
C	10.90166700	-3.37863400	1.27889100
C	11.38589300	-2.93098300	-0.08294800
O	10.30327500	-3.02523500	-0.97399300
C	10.57660800	-2.50892300	-2.25285500
C	9.27773200	-2.48825900	-3.02972200
O	8.45347300	-1.49338200	-2.47204000
C	7.08564500	-1.65747500	-2.74817600
C	6.32011300	-0.68127800	-1.88470100
O	6.55690400	-1.05732000	-0.53901800
C	6.08712500	-0.25551000	0.43833400
C	5.24203300	0.82573000	0.24088300
C	4.80820300	1.56652900	1.34628700
C	5.23475400	1.27055400	2.62655600
C	6.11189900	0.19881400	2.82248100
N	3.89727700	2.64746200	1.14710900
N	4.20632400	3.86603700	1.62118400
N	3.21935500	4.65383000	1.34826100
C	2.24172400	3.94875500	0.70845800
C	2.66328600	2.63267100	0.56636000
C	1.00075100	4.62262000	0.29014700
C	1.06621600	5.94085900	-0.17345500
N	-0.00114800	6.66116200	-0.52195700
C	-1.19572100	6.08068400	-0.42769200
C	-1.39321200	4.76865100	0.02264300
C	-0.26377200	4.03878400	0.39681100
C	-2.75794500	4.23980900	0.13330100
C	-3.24440000	2.94963000	-0.05003000
N	-4.57486600	3.09306500	0.20771000
N	-4.88954100	4.36999600	0.49655400
N	-3.79943300	5.05972100	0.45493400
C	-5.60579900	2.10825300	0.22016600
C	-5.41489400	0.93338000	0.95505900
C	-6.39968000	-0.04160300	0.94823000
C	-7.60655200	0.18100600	0.23939500
C	-7.78326300	1.36839400	-0.45913400
C	-6.77425600	2.33615500	-0.48105000
O	-8.50870000	-0.82268000	0.31024600
C	-9.65312000	-0.74578500	-0.51992200
C	-10.38089500	-2.06232900	-0.38782400
O	-9.59810900	-3.05839700	-1.00011800
C	-9.97922600	-4.37164000	-0.65229100
C	-9.36392200	-4.82602700	0.65868600
O	-7.97259900	-4.91562000	0.48754500
C	-7.30320500	-5.25925000	1.67503600
C	-5.80961000	-5.17222900	1.42091000

O	-5.38769400	-3.84830800	1.18029100
C	-5.12934800	-3.11755700	2.35418000
C	-5.01299800	-1.65808000	1.98575300
O	-6.30608100	-1.22770400	1.58826800
Te	1.72799700	1.00196100	-0.41931000
Te	-2.20691200	1.20815700	-0.69420500
H	8.60253900	-0.86551200	3.29539200
H	7.44939500	-2.11248300	3.85064400
H	9.66342300	-3.11809100	3.69182000
H	8.62292800	-3.79335600	2.41126400
H	11.75392500	-3.51186300	1.96280000
H	10.39394000	-4.34826400	1.17110100
H	12.22362900	-3.56823900	-0.40956300
H	11.75009600	-1.89405300	-0.02151500
H	11.32015900	-3.12976700	-2.77824200
H	10.97523500	-1.48545800	-2.18101600
H	9.47285000	-2.28860400	-4.09447000
H	8.80089900	-3.47599300	-2.94767800
H	6.85951400	-1.46278000	-3.80786800
H	6.76475100	-2.68241200	-2.51134200
H	6.68344200	0.34025300	-2.05714100
H	5.24796700	-0.72496000	-2.11779100
H	4.90601300	1.10107400	-0.75116100
H	4.88813100	1.86502200	3.46414600
H	6.45157700	-0.03289600	3.82497100
H	2.03471400	6.42899400	-0.25338400
H	-2.05723300	6.67854000	-0.71512800
H	-0.36100000	3.04107900	0.81693700
H	-4.49176000	0.79044500	1.50462600
H	-8.69989300	1.54769500	-1.00789200
H	-6.89823500	3.25643400	-1.04024200
H	-9.35452700	-0.58818300	-1.56427600
H	-10.30365400	0.08120200	-0.20553400
H	-11.36673200	-1.98925800	-0.87249500
H	-10.54153700	-2.27175500	0.67900100
H	-11.07451000	-4.46196800	-0.59846900
H	-9.78887500	-5.80371800	0.94185900
H	-7.56682400	-6.28254000	1.99046600
H	-7.59600300	-4.57621200	2.48860400
H	-5.57009600	-5.74607100	0.52116300
H	-5.26181300	-5.61276400	2.26732200
H	-4.19180800	-3.45298100	2.82295200
H	-5.93889200	-3.23393200	3.08907500
H	-4.30212400	-1.53163200	1.15806700
H	-4.66120200	-1.07907900	2.84962300
H	-9.61952000	-5.02345400	-1.45371100
H	-9.60298000	-4.11577500	1.46652900
C	-3.77975800	0.49813300	-1.97118200
C	2.08723300	-0.37741200	1.18385800
H	-3.28967700	-0.18995700	-2.66365800
H	-4.20556400	1.33978600	-2.51948800
H	-4.55134800	-0.02716800	-1.40845800
H	1.88834000	0.12055100	2.13385200
H	3.11140700	-0.75067300	1.15051200
H	1.37831300	-1.19369900	1.03148600

I	-0.31742700	-1.73497800	-1.79136100

[**1•XB** +2Na+I]$^{1+}$
100

C	-7.25967700	0.16271900	-0.79398500
O	-8.15090500	-0.85753000	-0.98157800
C	-9.47233600	-0.53415500	-1.41526700
C	-10.27017700	-1.82094200	-1.41066700
O	-10.22093900	-2.37257800	-0.10511900
C	-10.87475300	-3.62907500	0.01539300
C	-10.50180700	-4.21803600	1.35938600
O	-9.09418000	-4.40085400	1.36668400
C	-8.54907800	-4.77985300	2.62147800
C	-7.04099100	-4.72625800	2.49682200
O	-6.67861500	-3.38456200	2.20140900
C	-5.32592700	-3.22390500	1.80472800
C	-5.10949100	-1.76818700	1.44971800
O	-6.06550700	-1.44136700	0.44082200
C	-6.12509800	-0.15722600	-0.01772700
C	-5.16824400	0.80681300	0.24574400
C	-5.31830100	2.07980500	-0.31133400
C	-6.42472100	2.40705000	-1.07641600
C	-7.40802100	1.44254400	-1.30575100
N	-4.32657700	3.07218800	-0.07029100
N	-4.71066100	4.30510100	0.30754900
N	-3.65043800	5.02979800	0.42309000
C	-2.54672900	4.28487600	0.12416200
C	-2.97220500	3.00863500	-0.20420500
C	-1.20797800	4.89002200	0.14492800
C	-1.07747300	6.24335900	-0.18807800
N	0.08150400	6.89942300	-0.17855500
C	1.17552600	6.21687400	0.15406200
C	1.17344700	4.85944400	0.50051000
C	-0.05340700	4.19383400	0.50734800
C	2.44814900	4.23295900	0.87906000
C	2.92884400	2.94481000	0.71020500
N	4.19389500	3.00124900	1.21263800
N	4.47354700	4.23745800	1.66464400
N	3.43415400	4.97339900	1.46453700
C	5.18839100	1.98634100	1.31051900
C	5.50774400	1.22972700	0.17762700
C	6.46668400	0.24087100	0.28915300
C	7.13765700	0.03539200	1.51198300
C	6.82679100	0.81024000	2.61745100
C	5.83492400	1.79075200	2.51847500
O	8.06102100	-0.96644600	1.47472700
C	8.76386400	-1.30878400	2.66309700
C	9.75617100	-2.37441300	2.26051300
O	9.02853900	-3.46763400	1.72434400
O	8.79573900	-4.18448300	-0.94803100
C	8.82914700	-3.95883900	-2.34758000
C	7.38971100	-3.91033100	-2.82310000
O	6.66802900	-2.87469100	-2.16568600
C	6.74017900	-1.62027300	-2.82530400

C	6.10289400	-0.58325100	-1.93188700
O	6.84622000	-0.59836000	-0.71551500
I	-1.90394000	1.35267900	-0.88270400
I	2.02945400	1.23862000	-0.09127500
H	-9.90640400	0.20368500	-0.73056200
H	-9.45402300	-0.12423900	-2.43034800
H	-11.30532700	-1.60549400	-1.70348500
H	-9.84219600	-2.53227100	-2.13081100
H	-11.96200300	-3.50354900	-0.05477000
H	-10.54267400	-4.29905000	-0.78999800
H	-11.01691700	-5.17563900	1.50487800
H	-10.79403400	-3.53248900	2.16730800
H	-8.86676100	-5.79512800	2.88969600
H	-8.88898300	-4.08467900	3.40167000
H	-6.57186700	-5.05390600	3.43256200
H	-6.70966300	-5.38795800	1.68442800
H	-4.64456500	-3.50322800	2.61782700
H	-5.11489800	-3.86100400	0.93429700
H	-5.26934100	-1.12261200	2.32136000
H	-4.09101000	-1.63064800	1.07083600
H	-4.31604400	0.58861800	0.87698100
H	-6.52182700	3.40453800	-1.48783900
H	-8.27782400	1.70137900	-1.89727000
H	-1.96143300	6.81153700	-0.46657000
H	2.11452800	6.76454300	0.15353400
H	-0.11606100	3.15767200	0.82446600
H	5.00842200	1.42335700	-0.76319000
H	7.33116800	0.65253300	3.56337400
H	5.56790700	2.39506800	3.37714800
H	8.06048400	-1.68559300	3.41543700
H	9.29337600	-0.43643600	3.06132200
H	10.33412400	-2.69335800	3.13669800
H	10.44741200	-1.96366300	1.51263900
H	9.36494900	-4.77210000	-2.85421600
H	9.35210700	-3.01463700	-2.55980100
H	6.88864700	-4.84725700	-2.56631500
H	7.34942000	-3.77818100	-3.91055100
H	6.20403400	-1.66192200	-3.78116100
H	7.78560700	-1.34383900	-3.01799400
H	5.05293200	-0.82481200	-1.72594000
H	6.16559800	0.40133800	-2.40769900
C	10.07343400	-4.20675000	-0.33477000
H	10.69437200	-5.00124400	-0.76934000
H	10.58086300	-3.24382600	-0.49505500
C	9.84892500	-4.47432800	1.14102200
H	10.80752500	-4.54473200	1.66784400
H	9.31364600	-5.41977100	1.26079200
I	0.24508800	-1.38301800	-1.50571700
Na	7.31175400	-2.80255300	0.14151300
Na	-8.04673800	-2.54080900	0.57540700

[**1•ChB** +2Na+I]$^{1+}$
108

C	-7.31138900	0.21520600	-0.42062600

O	-8.16509400	-0.85333600	-0.42590500
C	-9.29994200	-0.82434100	0.44040700
C	-9.97404100	-2.17559400	0.33271200
O	-9.02835700	-3.16908000	0.68930200
C	-9.50399500	-4.50157000	0.55442000
C	-8.31980000	-5.43138400	0.71424200
O	-7.40062400	-5.12975300	-0.32435700
C	-6.15034500	-5.79457000	-0.22316400
C	-5.25290000	-5.23333000	-1.30534600
O	-5.10227900	-3.84229000	-1.06045500
C	-4.50592000	-3.12759500	-2.12831500
C	-4.48575000	-1.65933800	-1.76399100
O	-5.83956100	-1.25752900	-1.53318100
C	-6.05600700	-0.00577000	-1.02407000
C	-5.12554600	1.01777500	-1.07414800
C	-5.42564400	2.24679200	-0.48186600
C	-6.65520100	2.47613200	0.11199700
C	-7.60564500	1.45396900	0.13082700
N	-4.44088600	3.26991700	-0.49070900
N	-4.78436200	4.51897500	-0.85223400
N	-3.71981600	5.24449600	-0.79400100
C	-2.65992700	4.47185200	-0.41183400
C	-3.11196900	3.17730300	-0.18842300
C	-1.32249400	5.07030200	-0.28055300
C	-1.21785500	6.42306800	0.06613400
N	-0.06145900	7.07408300	0.18027900
C	1.05720900	6.38874200	-0.04998600
C	1.08403600	5.03797500	-0.41880900
C	-0.13817000	4.37434200	-0.53946500
C	2.39082400	4.40599800	-0.66500600
C	2.86804200	3.13860500	-0.35767900
N	4.16219300	3.19244000	-0.80504800
N	4.44808600	4.38825100	-1.34346300
N	3.38783800	5.12028100	-1.26448100
C	5.20277300	2.22632400	-0.70444500
C	4.93823600	0.88804600	-1.01293100
C	5.94562200	-0.04476300	-0.85494800
C	7.23116400	0.35654700	-0.44306500
C	7.48956700	1.69135200	-0.17681900
C	6.46290000	2.63266500	-0.29642200
O	8.12174000	-0.66980500	-0.32795500
C	9.44676900	-0.39583300	0.10896700
C	10.18420300	-1.71229300	0.02945000
O	9.51344300	-2.63651800	0.87096500
C	9.99637900	-3.97114700	0.76595200
C	9.23951200	-4.74317900	-0.29788800
O	7.87231400	-4.72611300	0.07517600
C	7.01861100	-5.43189000	-0.80947500
C	5.60728900	-5.27014200	-0.27752200
O	5.24408500	-3.89610000	-0.20736200
C	4.65071600	-3.40089200	-1.39652300
C	4.49508200	-1.90569500	-1.25578000
O	5.80617400	-1.38664800	-1.05983500
Na	7.09713800	-2.62440100	0.60815100
Na	-7.09452700	-2.88144100	-0.48131800

Te	-2.15782900	1.45039600	0.61933300
Te	1.93172700	1.53887300	0.69517100
H	-8.96781500	-0.63162400	1.46730600
H	-9.99973600	-0.04186900	0.12908400
H	-10.84051700	-2.20244100	1.00516800
H	-10.32233100	-2.34291500	-0.69636800
H	-10.26052100	-4.71723600	1.31856700
H	-9.95499500	-4.63675300	-0.43850800
H	-8.65111400	-6.47510300	0.64681700
H	-7.84434600	-5.27371400	1.69242400
H	-6.27484100	-6.87570800	-0.36179300
H	-5.70900200	-5.61257100	0.76660000
H	-4.27713300	-5.73375500	-1.28603600
H	-5.71340800	-5.38909700	-2.29085500
H	-3.47690800	-3.46845100	-2.29846100
H	-5.08536600	-3.28151300	-3.04981900
H	-3.89823500	-1.48850300	-0.85368000
H	-4.05311600	-1.08901300	-2.59257000
H	-4.17706100	0.88284300	-1.58158400
H	-6.86539300	3.43840600	0.56342900
H	-8.56606200	1.62968600	0.60035300
H	-2.12344200	6.99357900	0.25725600
H	1.99467100	6.93048900	0.05156600
H	-0.17013500	3.33307900	-0.85069400
H	3.95988100	0.59411300	-1.36802900
H	8.47261600	2.01098000	0.14815000
H	6.64252300	3.67588800	-0.06629700
H	9.42895700	-0.01434900	1.13709400
H	9.92314400	0.34122100	-0.54673200
H	11.22154500	-1.57858400	0.36011400
H	10.18681700	-2.06572100	-1.01037400
H	11.07156500	-3.97810500	0.55238400
H	9.61158500	-5.77498000	-0.34666400
H	7.29024000	-6.49526200	-0.83796200
H	7.11399400	-5.02308200	-1.82632600
H	5.55729100	-5.65618500	0.74387500
H	4.89525900	-5.82698000	-0.89759600
H	3.66693300	-3.86086100	-1.55035100
H	5.28486800	-3.62098900	-2.26582200
H	3.86504100	-1.64935000	-0.39397700
H	4.05152700	-1.49605900	-2.17050300
H	9.83066300	-4.43813900	1.74012100
H	9.36176900	-4.28345200	-1.28965600
C	1.35075000	0.59003800	-1.14851000
C	-1.37867200	2.58511900	2.26067300
H	1.68260800	-0.44923200	-1.14324000
H	1.80627800	1.13851900	-1.97644000
H	0.26330000	0.62364000	-1.23001500
H	-2.20477800	3.07343900	2.77797500
H	-0.65086100	3.31184800	1.89917400
H	-0.88765300	1.86327600	2.91652200
I	-0.04804600	-1.12833600	1.80498900
